# Simultaneous quantification of four antiretroviral drugs in breast milk samples from HIV-positive women by an ultra-high performance liquid chromatography tandem mass spectrometry (UPLC-MS/MS) method

**DOI:** 10.1371/journal.pone.0191236

**Published:** 2018-01-19

**Authors:** Alicia Ramírez-Ramírez, Elías Sánchez-Serrano, Giselle Loaiza-Flores, Noemí Plazola-Camacho, Rosa Georgina Rodríguez-Delgado, Ricardo Figueroa-Damián, Mauricio Domínguez-Castro, Margarita López-Martínez, Zayra Flores-García, Jessica Hernández-Pineda

**Affiliations:** 1 Departement of Infectology and Immunology, National Institute of Perinatology, Mexico City, Mexico; 2 Chemistry Faculty, National Autonomous University of Mexico, Mexico City, Mexico; 3 Department of Physiology and Cellular Development, National Institute of Perinatology, Mexico City, Mexico; 4 Faculty of Higher Education, National Autonomous University of Mexico, Cuautitlan Izcalli, Mexico State, Mexico; Istituto di Genetica Molecolare, ITALY

## Abstract

The primary strategy to avoid mother-to-child transmission of human immunodeficiency virus (HIV) through breastfeeding is administration of highly active antiretroviral therapy (HAART) to HIV-positive pregnant women. Because significant changes in the pharmacokinetics of antiretroviral (ARV) drugs occur during pregnancy, quantifying HAART and the viral load in breast milk in this population is essential. Here, we developed an analytical assay for the simultaneous quantification of four ARV drugs in breast milk using ultra-performance liquid chromatography coupled to tandem mass spectrometry. We validated this method following Mexican and international guidelines. ARV drugs. We extracted the ARV drugs from 200 μL samples of breast milk and detected these drugs in a triple quadrupole mass spectrometer with positive electrospray ionization. The validated concentration ranges (ng/mL) for zidovudine, lamivudine, lopinavir, and ritonavir were 12.5–750, 50–2500, 100–5000 and 5 to 250, respectively. Additionally, the absolute recovery percentages (and matrix effects) were 91.4 (8.39), 88.78 (28.75), 91.38 (11.77) and 89.78 (12.37), respectively. We determined that ARV drugs are stable for 24 h at 8°C and 24°C for 15 days at –80°C. This methodology had the capacity for simultaneous detection; separation; and accurate, precise quantification of ARV drugs in human breast milk samples according to Mexican standard laws and United States Food and Drug Administration guidelines.

## Introduction

Breastfeeding is the gold standard of infant nutrition. It reduces the rates of morbidity and death in childhood by avoiding diarrheal diseases and the risk of obesity. Breast milk is composed of complex proteins, lipids, and carbohydrates that improve the development of intestinal microbiota and the immune system in children [1]. However, lactation is associated with forty-two percent of the mother-to-child transmission (MTCT) cases of human immunodeficiency virus (HIV) [2]. Various interventions have been devised to prevent this type of transmission, and the most useful method proposed is the treatment of both mothers and neonates with ARV prophylaxis to prevent HIV infection [1, 2]. This treatment consists of three or four ARV drugs that constitute highly active ARV therapy (HAART) [1, 3]. The use of multiple ARV drugs increases treatment efficacy and improves the control of HIV disease progression compared to monotherapy [4, 5]. Currently, five ARV drug families with different mechanisms of action on the HIV life cycle are available. These include: 1) fusion inhibitors, which inhibit fusion between the virus and the lymphocyte cellular membrane; 2) nucleosides analogous to reverse transcriptase inhibitors (NRTIs) and 3) non-nucleoside reverse transcriptase inhibitors (NNRTIs), which both inhibit reverse transcriptase enzymatic function, introducing a stop signal into the viral DNA sequence preventing viral replication [3]; 4) integrase strand transfer inhibitors, which block HIV integrase enzyme function and replication; and 5) protease inhibitors (PIs), which block protease enzyme function in HIV and thus prevent the generation of immature HIV forms and their transformation into mature virus, with the ability to infect new cells [1, 3]. Most international therapy guidelines recommend a regimen of two NRTIs, in combination with one NNRTI or one PI, and reinforced with Ritonavir [6, 7]. In the particular case of pregnant women, HAART comprises one tablet of Combivir (zidovudine [ZDV]/lamivudine [LMV]; NRTI drugs) and two Kaletra tablets (lopinavir [LPV]/ritonavir [RTV]; PI drugs), both taken every 12 hours [8]. However, the choice of the most appropriate drug therapy in this population made by considering efficacy and, avoiding toxicity and teratogenic effects in neonates [1, 6]. The benefits of HAART in very low- resource countries must be balanced with the availability of funds. As a result, only 67% of HIV-positive pregnant women receive HAART [9, 10]. In Mexico, 43.2% of HIV-positive pregnant women receive these drugs [11]. Physiological changes that occur during pregnancy and lactation do not allow the distribution of ARV drugs evenly throughout the maternal body. These changes include increased progesterone, gastric pH, body fat, water content, and dilution space in the pregnant woman [12]. Because the level of plasmatic protein decreases, the transport of drugs is diminished, which increases the half-life of the active fraction (e.g., LPV/RTV) in blood [12, 13]. Elimination of ARV drugs is performed through hepatic metabolism and renal excretion. During pregnancy, the increase in progesterone and estrogens leads to inhibition or induction of some hepatic cytochrome P450 (CYP) isoenzymes, which increases or decreases drug clearance once the rate of renal filtration is also increased [13] The excretion of ARV drugs through breast milk decreases their effectiveness in patients, and the viral load does not decrease sufficiently [12,13,14]. Currently, there is not enough scientific evidence to correlate HIV load with changes in the pharmacokinetics of ARV drugs in plasma and breast milk during pregnancy [14,15]. Consequently, this issue remains controversial [2, 16]. Strict adherence to HAART among HIV-positive women could result in undetectable or very low viral loads in breast milk and would increase the opportunity for these women to breastfeed their children safely [1, 9]. The World Health Organization (WHO) guidelines recommend that mothers exclusively breastfeed their babies during the first six months of life; this includes HIV-positive women [15] while the neonate receives ARV monotherapy and the mother receives HAART [1, 7, 10]. Therefore, the ARV drug concentration in breast milk from HIV-positive women must be determined [17]. Hence, we focused on developing and validating a high-sensitivity, accurate method based on ultra-performance liquid chromatography tandem mass spectrometry (UPLC-MS/MS) method for the simultaneous quantification of ZDV, LMV, LPV and RTV in breast milk samples.

## Materials and methods

### Materials

The reference standards LMV, ZDV, LPV, and RTV and internal standard (IS) simvastatin (SMV) were purchased from the U.S. Pharmacopeia Convention (Rockville, MD, USA). Methanol (MeOH), acetonitrile (ACN), and 2-propanol (high-performance LC [HPLC] grade) were obtained from J.T. Baker (Center Valley, PA, USA). We used water from an 18.2-MΩ Millipore Synergy^®^ UV water purification system (Bedford, MA, USA). Formic acid was acquired from Fluka (Sigma-Aldrich Co., St. Louis, MO, USA) and was of LC-MS quality. Breast milk samples (colostrum, transition, and mature milk) from HIV-negative women were provided by the breast milk bank of the National Institute of Perinatology (INPer, Mexico City, Mexico).

### UPLC-MS/MS systems and conditions

Chromatographic separation was conducted using an ACQUITY UPLC H-Class System coupled to a Xevo TQ-S tandem mass detector equipped with an ESI source from Waters (Waters Corp., Milford, MA, USA). Data acquisition, peak integration, data processing, and reporting were performed with MassLynx version 4.1 software (Waters Corp.). Chromatographic separation was performed using an ACQUITY UPLC^®^BEH C18 column (2.1 mm × 50 mm ID; particle size: 1.7 μm; Waters Corp., Milford, MA, USA). The mobile phase consisted of 0.1% formic acid in water (phase A) and ACN (phase B) gradients, shown in Table 1. The assay run time was 2.5 min with a flow rate of 0.50 mL/min, separation was performed at 35°C, the autosampler temperature was 5°C and the injection volume was 3 μL. In the MS, to create precursor ions of each ARV, we used positive electrospray ionization. Data were acquired in multiple-reaction-monitoring (MRM) mode.

**Table 1:** Chromatographic conditions: Mobile phase gradient program.

Time (minutes)	% phase A	% phase B	Speed of gradient change (Curve type*)
0.15	90	10	2
0.70	60	40	2
2.0	10	90	6
2.5	90	10	11

*Curve 2: concave type. Curve 6: lineal type.

The capillary voltage was set at 2.9 kV, the vaporizer temperature was set at 150°C, and the collision gas was nitrogen at 50 L/hr. The cone voltage and collision energy were determined for each drug. Transition ions are shown in Table 2.

**Table 2:** Transition ions and optimal conditions to obtain relative abundance of product ions.

Parameter	LMV	ZDV	LPV	RTV	SMV
**Transition (m/z)** **Precursor ion →product ion**	230.15 → 112.08	268.18 → 127.10	629.55 → 447.35	721.50 → 296.20	419.40 → 199.20
**Dwell (s)**	0.161	0.161	0.105	0.105	0.105
**Cone Voltage (kV)**	15	10	20	23	20
**Collision energy (kV)**	15	10	15	15	15
**Total ion count (TIC)**	1.02e^7^	1.18e^6^	6.11e^6^	4.55e^6^	3.41e^5^

m/z: mass/charge relationship; kV: kilovolts.

### Stock solutions, working solutions, and quality control (QC) solutions

Stock solutions of LMV, ZDV, LPV, and RTV were produced at a final concentration of 1 mg/mL. The stock solutions of LPV and RTV were dissolved in ACN, whereas the stock solutions of LMV and ZDV were prepared in methanol/water (50/50). IS, the internal, had a final concentration of 1 mg/mL in methanol/water (50/50). We generated an intermediate working solution (a mixture of the four ARVs) from the stock solutions of each ARV drug using the following final concentrations: LMV = 6,000 ng/mL, ZDV = 1,500 ng/mL, LPV = 12,000 ng/mL, and RTV = 600 ng/mL. Seven-point calibration solutions were obtained by serial dilution, and the concentration ranges (ng/mL) were as follows: LMV = 60,000 to 1,000, ZDV = 15,000 to 250, LPV = 120,000 to 2,000, and RTV = 6,000 to 100. The working solution of the IS had a final concentration of 10,000 ng/mL in ACN.

Quality control (QC) solutions were created by diluting the intermediate solution of the mixture of ARVs with ACN to obtain high-QC (HQC), medium-QC (MQC), and low-QC (LQC) solutions. The concentrations (ng/mL) of the HQC solutions were as follows: LMV = 50,000, ZDV = 12,500, LPV = 100,000, and RTV = 5,000. The concentrations of the MQC solutions were as follows: LMV = 34,000, ZDV = 8,500, LPV = 68,000, and RTV = 3,400. Finally, the concentrations of the LQC solutions were as follows: LMV = 6,000, ZDV = 1,500, LPV = 12,000, and RTV = 600.

### Calibration curve and QC standards in breast milk samples

We prepared calibration curves and QC standards in the matrix using 200 μL of drug-free breast milk and adding 10 μL of ARV mixture for calibration or QC solutions. The nominal concentrations of all calibration curves and QC standards are shown in Table 3.

**Table 3:** Nominal concentrations of each calibration curve and quality control standards in breast milk samples.

POINTS	ZDV (ng/mL)	LMV (ng/mL)	LPV (ng/mL)	RTV (ng/mL)
**1**	12.5	50	100	5
**2**	25	100	200	10
**3**	50	200	400	20
**4**	125	500	1000	50
**5**	250	1000	2000	100
**6**	500	2000	4000	200
**7**	750	3000	6000	300
**LQC**	75	300	600	30
**MQC**	425	1700	3400	170
**HQC**	625	2500	5000	250

LQC: Low quality control standard. MQC: Medium quality control standard. HQC: High quality control standard

### Sample pretreatment

Ten microliters of the 10,000 ng/mL SMV working solution as an IS was added to the samples (calibration curve, QC standards and samples from HIV-positive women) and mixed for 1 min. All samples, were treated with hexane (100 μL) and frozen (-80°C) to extract the lipids. Subsequently, we precipitated the proteins with 0.1% formic acid in cold ACN (300 μL). Then, the samples were centrifuged at 10,000 rpm for 5 min at 5°C to produce the supernatant. Each sample was filtered through a 0.22-μm syringe filter and transferred to an autosampler vial.

### Calibration curves, accuracy, and precision

To determine the instrument response to calibration curve standard, these concentrations were calculated based on the previously reported maximum drug concentrations. The final concentration ranges (ng/mL) of each ARV in the breast milk samples were as follows: LMV = 3,000 to 50, ZDV = 750 to 12.5, LPV = 6,000 to 100, and RTV = 300 to 5. The chromatographic peaks were integrated valley to valley with a smooth 3x3 and with default parameters of Masslynx software. The calibration curves were analyzed using the ratio of the peak area of each ARV and IS and a 1/*x* weighted lineal regression. Next, we calculated the correlation coefficient (r) and the coefficient of determination (r^2^). Both should be close to 100%. We also determined the precision and accuracy for the QC standards of each ARV drug. Mexican quality standards require that absolute deviation (%AD) values do not exceed 15% for QC standards; the same requirements are specified for linearity parameters (i.e., the coefficient of variation [%CV]) [18]. In one working day, the precision was measured (n = 5) using the following standards: the lower limit of quantification (LLOQ) (i.e., a standard containing at least 5% of the maximum concentration value proposed for each drug), HQC, MQC, LQC, and diluted QC (DQC) (i.e., a standard containing 1.5× the highest concentration of each drug in the calibration curve, diluted 1:4 with drug-free breast milk). The mean, standard deviation (SD), and %CV of each ARV drug was determined. We measured the reproducibility (i.e., the precision on different working days) in the LLOQ, HQC, MQC, LQC, and DQC standards (n = 5) on three consecutive days. Then, we calculated the mean, SD, and %CV. The accuracy (i.e., concordance between experimental data and nominal data) was also determined (%AD). Mexican quality guidelines [19] specify that %CV and %AD should be less than 15%.

### Recovery, dilution integrity, and matrix effect

Using the HQC, MQC, and LQC standards, made with breast milk, we estimated the recovery percentages of four ARV drugs (n = 5). We compared the results to those obtained for solutions containing the same drug concentration but no breast milk. This value must be reproducible for each concentration level (i.e., each QC standard) with breast milk samples, and although the concordance will not be 100%, the method must be sufficiently replicable and accurate. Dilution integrity was assessed using a DQC standard containing the ARV drugs at concentrations 1.5× higher than the top boundaries of the curves and diluted 1:4 with breast milk and then processed. We assayed a DQC standard of each ARV drug five times. The %CV must be lower than 15%. Matrix effect determination was performed using six different breast milk samples (n = 3), including colostrum and transition milk samples. All samples were processed (i.e., subjected to protein and lipid extraction) as described above. We also analyzed a series of samples that, after being subjected to lipid and protein extraction, were combined with only the LQC and HQC solutions (n = 3) of the ARV drug and the IS solution. The normalized matrix factor (NMF) reflects the relationship between the ARV drug concentration in breast milk and the IS concentration in breast milk divided by the values obtained from the relationship of ARV concentration in solution to the IS in solution. Mexican quality guidelines specify that the %CV of NMF values should be less than 15% [19].

### Stability and reinjection reproducibility

We determined the stability of the four ARV drugs in storage conditions, in solution, and in breast milk samples before, and after sample processing (i.e., lipid and protein extraction) [19]. We calculated the recovered concentrations of ARV drugs using an HQC, MQC, and LQC standard in breast milk at different temperatures and storage times. We always prepared a fresh calibration curve before estimating the %AD with respect to the nominal value, which must be less than 15%. Short-term stability was measured by assaying each QC standard three times. We stored the first series of QC standards at room temperature (24°C), refrigerated the second series (5°C), and froze the third series (–84°C). All series were stored for 24 hours. Subsequently, these samples were processed and analyzed by chromatography. Long-term stability tests were performed using frozen QC standards after storage for 15 days. After storage, the samples were thawed, the proteins and lipids were extracted, and the samples were analyzed by chromatography. Similarly, to establish the stability of the samples in the autosampler of the chromatography system, we stored some samples at 5°C for 24 hours. Additionally, we assayed the stability of the samples during three freeze-thaw cycles. We then, we extracted the lipids and proteins from samples before analyzing them by chromatography. Finally, the long-term stability of each ARV drug and the IS was determined after storage for one, two, and four weeks at 0°C. Only QC standards were subjected to this analysis. A standard was considered stable if the %AD of the analytical response was below 10%.

### Application of ARV drugs concentrations in breast milk

The method developed and validated here was applied to determine the breast milk levels of ARV in nine samples from four HIV-positive women. We obtained every colostrum sample from the patient by hand. These patients received the HAART regimen with at least two of the ARV studied here, during second or third trimester of pregnancy. All received, as prophylaxis treatment, LPV/RTV (200/150 mg) and ZDV/LMV (300/50 mg), with three doses every three hours, before the surgical procedure (cesarean section). The nine enrolled patients signed an informed consent form. The study protocol and breast milk sample collection were approved under project number (478) 212250–3120771 by the Research and Ethics Committee of the Instituto Nacional de Perinatología, Mexico City, Mexico.

Co-infections, including syphilis candidiasis and cysticercosis, and comorbidities such as gestational diabetes, arterial hypertension, colitis, arrhythmias and premature rupture of membranes were reported. We also reported the use of other ARVs such as tenofovir, emtricitabine, abacavir, efavirenz and raltegravir. CD4+ lymphocytes count and viral load were determined in blood samples.

Linearity, precision and accuracy, carryover, reagents blanks and the assessment of the suitability of the systems (adequacy), were also determined on the day of milk sample processing (S1 Supporting information report).

## Results

### LC-MS/MS conditions

**Fig 1:**
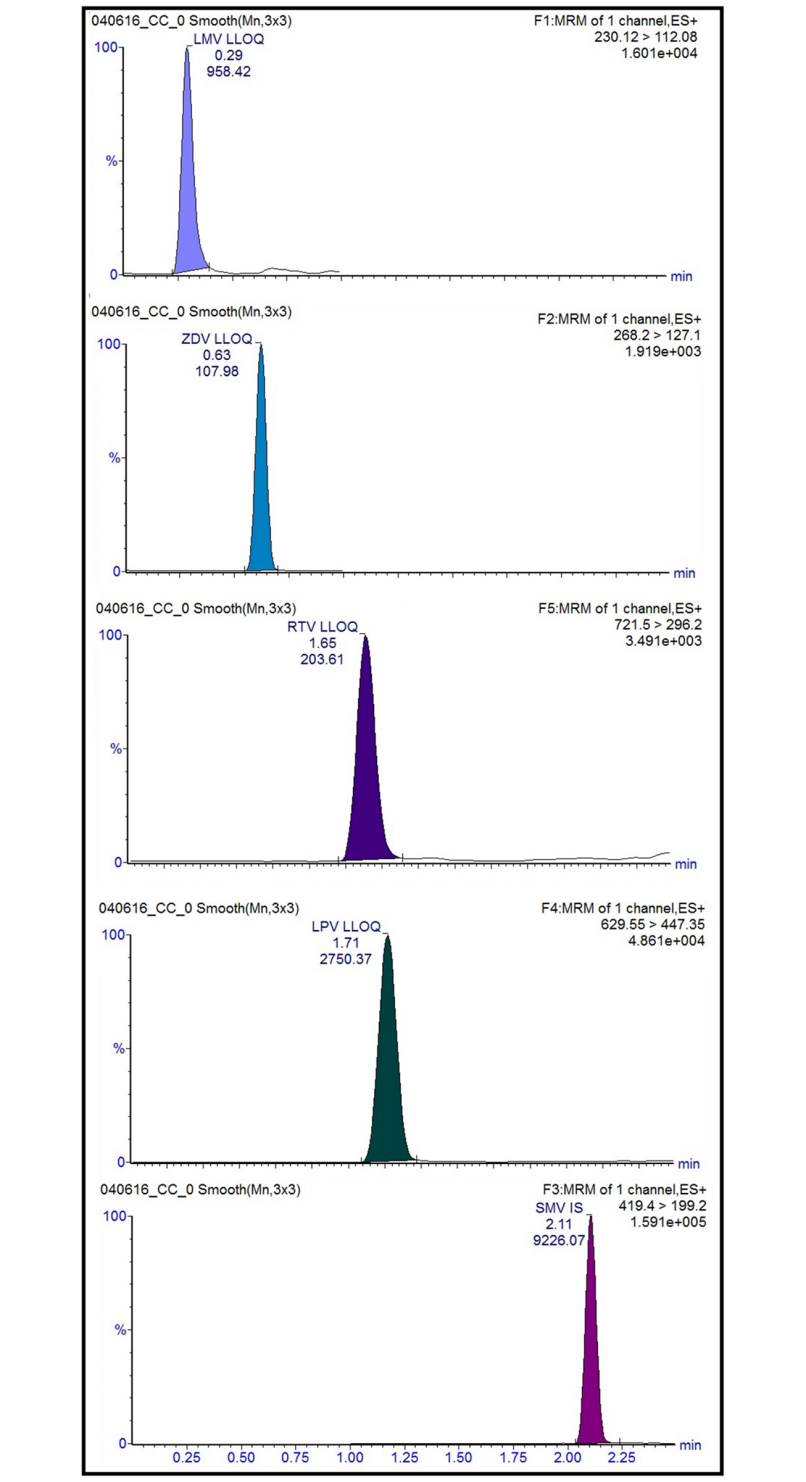
Representative ion chromatograms of separation and retention time (minutes) of each ARV drug and IS: 0.29, 0.62, 1.61, 1.71 and 2.11 for LMV, ZDV, RTV, LPV, and SMV, respectively, corresponding only to LQC (ng/mL) of each ARV. Total run time was 2.5 minutes, with an ACQUITY UPLC BEH C 18 column, formic acid and ACN gradient as mobile phase.

Transition ions from HAART drugs are shown in Table 2, as are the optimal conditions for obtaining a specific fragment of every ARV drug molecule and maximizing the numbers of these ions. Retention times (minutes) and some chromatograms are presented in Fig 1. Representative chromatograms for different QC standards of every ARV are shown in supporting figures (S1 to S4 Figs).

### Calibration curves, accuracy, and precision

The determination coefficient (r^2^) means of the calibration curves were 0.9982, 0.9993, 0.9995, and 0.9979 for ZDV, LMV, LPV, and RTV, respectively.

**Fig 2:**
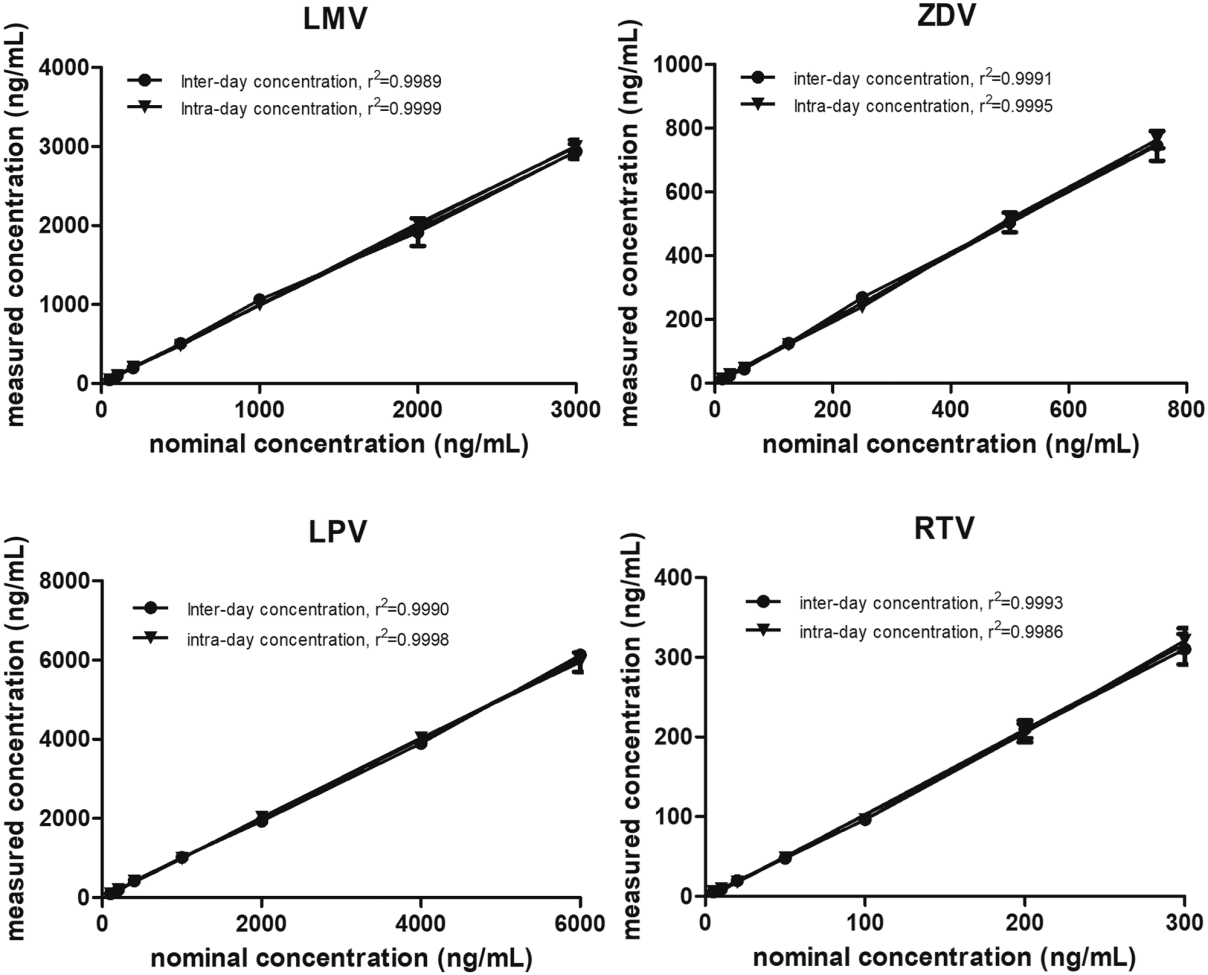
Correlation between nominal concentration *vs* measured concentration (ng/mL) for intra-day (n = 3) and inter-day (n = 3) validation of each ARV.

Method validation was conducted over a linear range between 12.5 and 750 ng/mL for ZDV, 50 and 2,500 ng/mL for LMV, 100 and 5,000 ng/mL for LPV, and 5 and 250 ng/mL for RTV. The correlations between nominal concentration vs measured concentration (ng/mL) are show in Fig 2 for intra-day (n = 3) and inter-day (n = 3) validation and S1 Table. The precision and accuracy percentages for the quantification of these concentrations of ARV drugs in breast milk standards are listed in Table 4.

**Table 4:** Accuracy and precision for the quantification of antiretroviral in breast milk.

QCs (ng/mL)	Inter-day (measured concentration)				Intra-day (measured concentration)			
	Mean	SD	Precision (%CV)	Accuracy (%)	Mean	SD	Precision (%CV)	Accuracy (%)
**ZDV (nominal concentration)**								
**75 (LQC)**	72.62	7.63	10.51	96.84	74.16	8.54	11.52	91.86
**425 (MQC)**	408.20	56.72	13.89	89.05	435.47	30.16	6.91	94.45
**625 (HQC)**	609.40	53.37	8.75	92.61	596.87	38.24	6.40	92.61
**LMV (nominal concentration)**								
**300 (LQC)**	307.41	17.75	5.77	94.86	309.18	22.76	7.36	93.99
**1700 (MQC)**	1761.26	217.89	12.37	88.02	1841.41	120.57	6.54	90.10
**2500 (HQC)**	2562.23	232.23	9.06	92.14	2611.72	109.65	4.19	93.84
**LPV (nominal concentration)**								
**600 (LQC)**	647.52	74.27	11.47	89.70	619.84	27.47	4.43	95.11
**3400 (MQC)**	3847.57	266.31	6.92	86.84	3689.62	212.61	5.76	91.49
**5000(HQC)**	5942.90	603.76	10.15	***81*.*15***	5610.38	232.20	4.13	86.69
**RTV (nominal concentration)**								
**30 (LQC)**	30.20	2.99	9.91	92.38	29.66	0.94	3.17	97.74
**170 (MQC)**	194.26	19.55	10.06	85.22	193.23	10.13	5.24	86.33
**250 (HQC)**	307.35	31.32	10.19	***77*.*06***	296.34	11.28	3.80	***81*.*23***

LQC: Low quality control standard. MQC: Medium quality control standard. HQC: High quality control standard

All concentrations comply with Mexican quality standards [19] and FDA guidelines for accuracy, except the HQC standards for RTV and LPV.

### Recovery, dilution integrity, and matrix effect

The recovery percentages of each ARV drug from breast milk samples after lipid and protein extraction (%CV) were 88.78%, 91.4%, 91.38%, and 89.78% for LMV, ZDV, LPV, and RTV, respectively. We evaluated the integrity of the DQC (1:4) standard and measured the %CV values of repeatability and reproducibility (shown in parentheses): 95.95 (73.27), 94.47 (69.06), 97.26 (68.97), and 96.79 (73.99) for LMV, ZDV, LPV, and RTV, respectively. In the same order, the %AD accuracy values for the DQC standards were: 77.27, 72.49, 71.57, and 75.89. The mean NMFs and their %CV values (shown in parentheses) were 0.597 (28.75%), 1.055 (8.39%), 0.559 (11.77%), and 0.575 (12.37%) for LMV, ZDV, LPV, and RTV, respectively.

### Stability and reinjection reproducibility

We assayed the short-term stabilities of LMV, ZDV, LPV, and RTV in processed (i.e., after the extraction of lipids and proteins) and unextracted (i.e., without the extraction of lipids and proteins) breast milk samples. We selected temperatures at room temperature (23°C), under refrigeration (5°C), and frozen after 24 hours, which within acceptable values (% precision and accuracy), as shown in Table 5. The MQC of ZDV and LQC of RTV standards at room temperature and the LQC standard of RTV at freezing temperatures failed to comply with Mexican standard criteria. A long-term stability assay of unextracted samples was performed by subjecting the samples to three freeze-thaw cycles separated by 12 hours each. The precision and accuracy were not valid for the LQC, MQC, and HQC standards of LPV according to Mexican standards.

**Table 5:** Short-term stability of antiretroviral drugs in processed samples.

Storage condition	Percentage stability								
	QC level	ZDV		LMV		LPV		RTV	
		Precision (%CV)	Accuracy (%)	Precision (%CV)	Accuracy (%)	Precision (%CV)	Accuracy (%)	Precision (%CV)	Accuracy (%)
**Stability of extracted samples (24 h at 23°C)**	LQC	4.87	95.50	2.63	90.00	4.30	95.72	4.49	89.69
	MQC	2.75	95.83	9.88	91.35	3.43	92.92	2.90	91.25
	HQC	3.18	94.68	2.78	94.16	7.90	94.43	6.67	90.83
**Auto sampler stability of extracted samples (24 h at 5°C)**	LQC	9.11	91.10	3.33	89.86	8.82	92.27	9.73	86.07
	MQC	4.64	91.24	3.84	91.40	3.79	88.37	4.45	86.68
	HQC	9.19	85.65	4.98	89.81	7.24	87.29	6.56	**77.29**
**Stability of unextracted samples (24 h at 23°C)**	LQC	2.02	95.96	1.33	98.00	4.59	85.64	25.93	**70.54**
	MQC	7.91	**83.12**	1.46	97.08	4.15	95.34	4.97	96.12
	HQC	1.85	88.56	1.27	96.69	3.40	95.54	1.59	95.07
**Stability of unextracted samples (24 h at 8°C)**	LQC	15.00	88.23	1.90	94.36	6.86	94.32	6.33	91.18
	MQC	1.39	94.88	1.19	96.52	3.55	91.06	4.42	97.00
	HQC	6.20	92.97	3.04	96.68	4.38	93.73	2.74	93.10
**Stability of unextracted samples (24 h at -84°C)**	LQC	7.08	95.37	2.37	94.26	0.83	85.69	1.60	**79.65**
	MQC	2.11	97.41	1.90	95.95	1.27	89.77	4.55	96.31
	HQC	2.08	96.73	3.10	97.73	2.90	89.54	5.68	95.70
Long-term stability of unextracted samples*	LQC	8.25	87.04	2.58	96.81	5.51	**71.74**	7.82	94.20
	MQC	4.87	90.29	4.11	96.74	9.44	**76.49**	9.44	91.99
	HQC	4.54	89.79	1.09	94.98	5.21	**44.55**	4.02	88.17
Long-term stability of unextracted samples**	LQC	2.35	**81.39**	1.04	93.56	2.18	**79.81**	1.10	89.27
	MQC	3.89	90.31	1.23	98.45	7.00	89.86	2.19	91.90
	HQC	4.75	87.57	2.91	97.37	2.36	**84.69**	4.90	**79.85**

*After three freeze-thaw cycles at -84°C.

**After 15 days at -84°C.

Attempts to quantify ARV drugs in breast milk samples after long-term storage in a freezer (i.e., 15 days) were unsuccessful, mainly using the QC standards of LPV. The LQC of ZDV and the HQC of RTV standards also failed to comply with Mexican standard criteria for long-term stability. We observed that ARV drugs kept at 5°C in the autosampler of the chromatography system for 24 hours were stable (Table 5).

### Clinical application: Quantitation of ARVs in breast milk from HIV-positive women using HAART

The mean age of the nine patients was 30 years (SD: ±5.14), the mean weight was 68.99 kg (SD: ±14.88), and the mean height was 157.25 cm (SD: ±6.06). The average gestational age at the time of delivery was 37.96 weeks (SD: ±0.81). The mean time after childbirth of breast milk collection was 25 hours (SD: ±15.24). The mean CD4+ lymphocyte count was 647.71 cells/μL (SD: ±467.04 cells/μL) (S2 Table). The average concentration (%CV) of LMV, ZDV, LPV, and RTV in breast milk samples was 448.83 ng/mL (73.48%), 100.66 ng/mL (132.56%), 4262.91 ng/mL (102.89%), and 239.56 ng/mL (56.31%), respectively. With this method, we detected all four ARVs in these samples. We found higher concentrations of IPs in two patients (S5 Fig and S3 Table).

## Discussion

Here, we described a UPLC-MS/MS method for the simultaneous quantification of multiple ARV drugs in 200-μL breast milk samples (colostrum, transition, or mature milk) that was successfully validated, with very few exceptions, using Mexican and International standards. ARV drugs have been quantified in plasma samples using immunoassays, HPLC-UV, and UPLC-UV methods [17, 18], which require significant biological sample volumes and are all time-consuming and laborious. In contrast to the methods mentioned above, this UPLC-MS/MS method can analyze many drugs simultaneously in a single sample [7, 8, 16, 18], even when the chemical characteristics of the drugs have marked differences in polarity and solubility. UPLC technology with ESI-MS/MS detection, allows simultaneous quantification of vastly different drugs in a short time. The sample volume is advantageous because of the difficulty of obtaining breast milk samples from HIV-positive women, who must avoid lactation. Among these women, as soon as birth occurs, healthcare workers inhibit maternal milk production. As a result, the opportunity to collect these samples is very brief, and sample volumes are minimal. The LQC of every ARV complained of the quality standard of accuracy and precision. Our objective is not to find a method to detect ARVs at deficient concentrations such as picograms per microliter (pg/μL). Rather, the aim was to establish a useful tool to determine therapeutic levels of ARVs in breast milk from HIV-positive women, in the stationary phase of ARV pharmacokinetics. Specifically, it was intended for patients using ZDV, LMV, RTV, and LPV as a HAART scheme, which is the most recommended during pregnancy in Mexico. Even when other drugs exist, such as last-generation ARVs (efavirenz), these four have demonstrated effectiveness and safety for the fetus, during pregnancy, ensuring no-vertical transmission of HIV and no teratogenic effects. At our institution, the prophylactic scheme before cesarean section always employs ZDV, LMV, RTV, and LPV (doses described in materials section). Therefore, our development focused on these ARVs.

The right choice of MS conditions, technology criteria for the column, the IS, and the methodology for the pretreatment of the sample, were, together, critical points to avoid interference and assure precision in every measurement.

The chemical conditions used to develop a useful extraction procedure applied to these samples were complicated of the complex composition of breast milk and because two of the ARV drugs were polar, and the other two were nonpolar. Thus, a precipitation reaction was selected carefully to avoid altering their characteristics and thus, the measurements of the four different drugs in the breast milk samples [17, 18]. Olagunju et al. [20], described the use of a paper-based method for analysis of a dry breast milk spot. In that study, the authors observed that breast milk samples were highly stable over a period of months. However, an elaborate chemical extraction procedure is required to elute the ARV drugs from the paper, which is a disadvantage of this technique that increases the process time needed for quantification. Additionally, with a poorly executed spotting method, the content of drug in the sample could be significantly underestimated [20, 21]. In contrast, our method utilizes an inexpensive and easy-to-perform procedure for the chemical extraction and precipitation of lipids and proteins from samples, thereby saving time and avoiding excessive sample handling by the analyst.

The simplicity of the method developed here and the use of reagents and materials commonly found in chromatography laboratories allow a secure and reliable methodology for routine studies of the HAART scheme for HIV-positive women and could complete our knowledge of the pharmacokinetics of these drugs in this population.

Using HAART, even with a reduced number of breast milk samples from HIV-positive women, we detected the excretion of both types of ARVs, NRTIs, and IPs, in these samples. We were able to elaborate on these results based on the high concentrations of ARVs administered to these women before cesarean section. In one study [12], the author suggested that the excretion of the IPs into breast milk are not easy, but we detected LPV and RTV in all breast milk samples, with, higher levels of these IPs in two samples. The amount of each ARV excreted by patients in this study showed high inter-individual variability. Therefore, we suggest additional studies to clarify the correlation between plasma ARV drug concentrations and excretion in breast milk. Additionally, the physiological changes during pregnancy and lactation should be studied to determine how they affect this concentration of drugs to find optimal doses of HAART and its relationship to the decreased viral load in these patients. The purpose of this project or the motivation to analyze this biological matrix was not to affirm that breastfeeding, in this population could be safe. Nor is prophylaxis treatment with breastfeeding a strategy for avoiding transmission to HIV-positive women’s children since we do not yet understand the pharmacokinetics of these or other drugs in this type of patient [22].

## Conclusions

Our study aimed to develop a robust LC-MS/MS methodology to achieve simultaneous quantification of four ARV drugs in breast milk samples from HIV-positive women. This development is inexpensive, useful, rigorously validated, recommended for routine study procedures. Currently, in INPer (Mexico City), this methodology is a relevant for two projects. One involves therapeutic drug monitoring, and the second population pharmacokinetics in pregnant HIV-positive women, with the objectives of personalized treatment for this community in our Institute, principally to prevent, underdosing with ARV drugs during pregnancy and prevent transmission of disease. In Mexico, HIV-positive women are not allowed to nurse their babies, even though the WHO has established that breastfeeding is a human right and highly recommended [15] for all new-born children, including from HIV-positive women. More research is needed on, regarding the use of specific regimens and their maternal and infant side-effect profiles in our population and with the development of validated analytical tools can be achieved these analyze.

## Supporting information

S1 Supporting Information Report Report of the linearity, precision and accuracy on the day of milk sample processing. (PDF)

S1 Fig Representative chromatograms of LMV extracted from breast milk. A) Blank with IS; B) LLOQ of LMV with IS; C) LQC of LMV with IS; D) MQC of LMV with IS; E) HQC of LMV with IS; and F) Blank of breast milk (.TIF).
(PDF)

S2 Fig Representative chromatograms of ZDV extracted from breast milk. A) Blank with IS; B) LLOQ of ZDV with IS; C) LQC of ZDV with IS; D) MQC of ZDV with IS; E) HQC of ZDV with IS; and F) Blank of breast milk (.TIF).
(PDF)

S3 Fig Representative chromatograms of LPV extracted from breast milk. A) Blank with IS; B) LLOQ of LPV with IS; C) LQC of LPV with IS; D) MQC of LPV with IS; E) HQC of LPV with IS; and F) Blank of breast milk (.TIF).
(PDF)

S4 Fig Representative chromatograms of RTV extracted from breast milk. A) Blank with IS; B) LLOQ of RTV with IS; C) LQC of RTV with IS; D) MQC of RTV with IS; E) HQC of RTV with IS; and F) Blank of breast milk (.TIF).
(PDF)

S5 Fig Representative chromatograms of separation and retention time (minutes) of each ARV drug and internal standard from two HIV-positive woman breast milk sample: 0.29, 0.64, 1.69, 1.74 and 2.16 for LMV, ZDV, RTV, LPV, and SMV. Total run time was 2.5 minutes, with an ACQUITY UPLC BEH C 18 column, formic acid and ACN gradient as mobile phase (.TIF).
(PDF)

S1 Table Accuracy and precision for quantification of antiretroviral in breast milk. (PDF)

S2 Table Demographics and clinical and ART individual data of HIV-positive women included in this study from National Institute of Perinatology, Mexico City, Mexico. (PDF)

S3 Table Antiretroviral levels in breast milk samples from HIV-positive women. (PDF)
