# Supplementary material for: Simultaneous quantification of four antiretroviral drugs in breast milk samples from HIV-positive women by an ultra-high performance liquid chromatography tandem mass spectrometry (UPLC-MS/MS) method
Source: PLoS One. 2018 Jan 19;13(1):e0191236. doi: 10.1371/journal.pone.0191236 (PMC5774716; doi:10.1371/journal.pone.0191236)
Supplement: S1 Supporting Information Report — (PDF) [file pone.0191236.s001.pdf]

Quantify Compound Summary Report MassLynx 4.1 SCN 843
Cuantificación de Antiretrovirales en Leche Materna

Dataset: C:\MassLynx\MAHM_13Jul15.PRO\LINEALITY_ANALYSIS_11302017.qld

Last Altered: Thursday, November 30, 2017 15:04:17 Central Standard Time (Mexico)
Printed: Thursday, November 30, 2017 15:07:55 Central Standard Time (Mexico)

Method: C:\MassLynx\MAHM_13Jul15.PRO\MethDB\071117_ZFG_NUEVO.mdb 30 Nov 2017 14:56:50
Calibration: 30 Nov 2017 15:04:17

Compound name: LMV
Correlation coefficient: r = 0.999074, r^2 = 0.998148
Calibration curve: 0.0012278 * x + 0.0217883
Response type: Internal Std (Ref 5), Area * (IS Conc. / IS Area)
Curve type: Linear, Origin: Exclude, Weighting: 1/x, Axis trans: None

	Name	Sample Text	RT	Height	Area	Acq.Date	Acq.Time	IS Area	%Dev
1	151117_STD_1	Lower Limit of Q...	0.289	30135	1115	15-Nov-17	18:11:21	13203.555	2.1
2	151117_STD_2	Standard 2	0.286	47420	1771	15-Nov-17	18:14:50	12271.620	-0.2
3	151117_STD_3	Standard 3	0.286	88423	3236	15-Nov-17	18:18:18	11723.644	3.5
4	151117_STD_4	Standard 4	0.286	208366	7776	15-Nov-17	18:21:48	13359.588	-8.7
5	151117_STD_5	Standard 5	0.286	426443	15863	15-Nov-17	18:25:13	12602.851	0.7
6	151117_STD_6	Standard 6	0.286	835852	31139	15-Nov-17	18:28:39	11994.957	4.8
7	151117_STD_7	Upper Limit of Q...	0.286	1184885	45017	15-Nov-17	18:32:05	12430.945	-2.3
8	151117_Blank_22	Blank	0.289	137	5	15-Nov-17	18:50:33	38.652	
9	151117_ QC_A_1	Quality Control 1	0.286	124315	4562	15-Nov-17	18:53:59	12167.331	-4.1
10	151117_ QC_B_1	Quality Control 1	0.286	799126	29599	15-Nov-17	18:57:25	12087.120	16.3
11	151117_ QC_C_1	Quality Control 1	0.286	921890	34881	15-Nov-17	19:00:53	11556.002	-2.4
12	151117_S_1	SAMPLE	0.286	195317	7405	15-Nov-17	19:07:48	10489.766	
13	151117_S_2	SAMPLE	0.289	73562	3223	15-Nov-17	19:11:13	8039.864	
14	151117_S_3	SAMPLE	0.286	60802	2348	15-Nov-17	19:14:39	11879.370	
15	151117_S_4	SAMPLE	0.289	28931	1205	15-Nov-17	19:18:06	4767.146	
16	151117_S_5	SAMPLE	0.286	43224	1810	15-Nov-17	19:21:33	7595.172	
17	151117_S_6	SAMPLE	0.283	73968	2692	15-Nov-17	19:25:00	7552.594	
18	151117_ QC_A_2	Quality Control 2	0.286	147546	5437	15-Nov-17	19:31:54	12742.463	9.9
19	151117_ QC_B_2	Quality Control 2	0.286	772311	28608	15-Nov-17	19:35:19	11741.281	15.7
20	151117_ QC_C_2	Quality Control 2	0.286	964397	35676	15-Nov-17	19:38:47	11770.134	-2.0
21	151117_ QC_A_3	Quality Control 3	0.286	147004	5378	15-Nov-17	21:29:37	12417.033	11.7
22	151117_ QC_B_3	Quality Control 3	0.286	801476	29555	15-Nov-17	21:33:04	12984.740	8.0
23	151117_ QC_C_3	Quality Control 3	0.286	1061063	39277	15-Nov-17	21:36:32	12252.269	3.7

Quantify Compound Summary Report MassLynx 4.1 SCN 843
Cuantificación de Antiretrovirales en Leche Materna

Dataset: C:\MassLynx\MAHM_13Jul15.PRO\LINEALITY_ANALYSIS_11302017.qld

Last Altered: Thursday, November 30, 2017 15:04:17 Central Standard Time (Mexico)
Printed: Thursday, November 30, 2017 15:07:55 Central Standard Time (Mexico)

Compound name: ZDV
Correlation coefficient: r = 0.999649, r^2 = 0.999298
Calibration curve: 0.000901537 * x + 0.00453434
Response type: Internal Std (Ref 5), Area * (IS Conc. / IS Area)
Curve type: Linear, Origin: Exclude, Weighting: 1/x, Axis trans: None

	Name	Sample Text	RT	Height	Area	Acq.Date	Acq.Time	IS Area	%Dev
1	151117_STD_1	Lower Limit of Q...	0.632	3542	210	15-Nov-17	18:11:21	13203.555	0.8
2	151117_STD_2	Standard 2	0.636	5813	339	15-Nov-17	18:14:50	12271.620	2.6
3	151117_STD_3	Standard 3	0.636	9915	593	15-Nov-17	18:18:18	11723.644	2.2
4	151117_STD_4	Standard 4	0.630	25112	1477	15-Nov-17	18:21:48	13359.588	-5.9
5	151117_STD_5	Standard 5	0.630	48488	2837	15-Nov-17	18:25:13	12602.851	-2.1
6	151117_STD_6	Standard 6	0.630	95851	5610	15-Nov-17	18:28:39	11994.957	2.7
7	151117_STD_7	Upper Limit of Q...	0.630	145540	8430	15-Nov-17	18:32:05	12430.945	-0.4
8	151117_Blank_22	Blank	0.621	68	6	15-Nov-17	18:50:33	38.652	
9	151117_ QC_A_1	Quality Control 1	0.630	14073	821	15-Nov-17	18:53:59	12167.331	-6.9
10	151117_ QC_B_1	Quality Control 1	0.633	97510	5720	15-Nov-17	18:57:25	12087.120	22.3
11	151117_ QC_C_1	Quality Control 1	0.630	128308	7420	15-Nov-17	19:00:53	11556.002	13.2
12	151117_S_1	SAMPLE	0.627	7307	496	15-Nov-17	19:07:48	10489.766	
13	151117_S_2	SAMPLE	0.630	2354	166	15-Nov-17	19:11:13	8039.864	
14	151117_S_3	SAMPLE	0.630	18944	1132	15-Nov-17	19:14:39	11879.370	
15	151117_S_4	SAMPLE	0.627	6461	399	15-Nov-17	19:18:06	4767.146	
16	151117_S_5	SAMPLE	0.619	2412	170	15-Nov-17	19:21:33	7595.172	
17	151117_S_6	SAMPLE	0.630	51045	3083	15-Nov-17	19:25:00	7552.594	
18	151117_ QC_A_2	Quality Control 2	0.630	17631	1021	15-Nov-17	19:31:54	12742.463	11.8
19	151117_ QC_B_2	Quality Control 2	0.630	92399	5402	15-Nov-17	19:35:19	11741.281	18.9
20	151117_ QC_C_2	Quality Control 2	0.630	118452	6969	15-Nov-17	19:38:47	11770.134	4.3
21	151117_ QC_A_3	Quality Control 3	0.630	17424	1021	15-Nov-17	21:29:37	12417.033	14.9
22	151117_ QC_B_3	Quality Control 3	0.630	95631	5575	15-Nov-17	21:33:04	12984.740	10.9
23	151117_ QC_C_3	Quality Control 3	0.630	128833	7412	15-Nov-17	21:36:32	12252.269	6.6

Quantify Compound Summary Report MassLynx 4.1 SCN 843
Cuantificación de Antiretrovirales en Leche Materna

Dataset: C:\MassLynx\MAHM_13Jul15.PRO\LINEALITY_ANALYSIS_11302017.qld

Last Altered: Thursday, November 30, 2017 15:04:17 Central Standard Time (Mexico)
Printed: Thursday, November 30, 2017 15:07:55 Central Standard Time (Mexico)

Compound name: LPV
Correlation coefficient: r = 0.998813, r^2 = 0.997627
Calibration curve: 0.00408171 * x + 0.163825
Response type: Internal Std (Ref 5), Area * (IS Conc. / IS Area)
Curve type: Linear, Origin: Exclude, Weighting: 1/x, Axis trans: None

	Name	Sample Text	RT	Height	Area	Acq.Date	Acq.Time	IS Area	%Dev
1	151117_STD_1	Lower Limit of Q...	1.729	127910	7346	15-Nov-17	18:11:21	13203.555	-3.8
2	151117_STD_2	Standard 2	1.726	198512	11341	15-Nov-17	18:14:50	12271.620	-6.9
3	151117_STD_3	Standard 3	1.729	394836	22666	15-Nov-17	18:18:18	11723.644	8.4
4	151117_STD_4	Standard 4	1.727	1315563	75313	15-Nov-17	18:21:48	13359.588	34.1
5	151117_STD_5	Standard 5	1.727	1858849	106220	15-Nov-17	18:25:13	12602.851	1.2
6	151117_STD_6	Standard 6	1.727	3628109	208046	15-Nov-17	18:28:39	11994.957	5.2
7	151117_STD_7	Upper Limit of Q...	1.726	5144194	293792	15-Nov-17	18:32:05	12430.945	-4.2
8	151117_Blank_22	Blank	1.729	1979	118	15-Nov-17	18:50:33	38.652	
9	151117_ QC_A_1	Quality Control 1	1.721	638086	36348	15-Nov-17	18:53:59	12167.331	15.3
10	151117_ QC_B_1	Quality Control 1	1.723	3525406	201667	15-Nov-17	18:57:25	12087.120	19.0
11	151117_ QC_C_1	Quality Control 1	1.723	4631191	264668	15-Nov-17	19:00:53	11556.002	11.4
12	151117_S_1	SAMPLE	1.727	4517936	257261	15-Nov-17	19:07:48	10489.766	
13	151117_S_2	SAMPLE	1.727	2768483	157903	15-Nov-17	19:11:13	8039.864	
14	151117_S_3	SAMPLE	1.727	72101	4135	15-Nov-17	19:14:39	11879.370	
15	151117_S_4	SAMPLE	1.723	872329	49831	15-Nov-17	19:18:06	4767.146	
16	151117_S_5	SAMPLE	1.721	1565441	89461	15-Nov-17	19:21:33	7595.172	
17	151117_S_6	SAMPLE	1.721	686331	39300	15-Nov-17	19:25:00	7552.594	
18	151117_ QC_A_2	Quality Control 2	1.727	664291	37870	15-Nov-17	19:31:54	12742.463	14.7
19	151117_ QC_B_2	Quality Control 2	1.727	3621858	206388	15-Nov-17	19:35:19	11741.281	25.5
20	151117_ QC_C_2	Quality Control 2	1.727	4659396	265916	15-Nov-17	19:38:47	11770.134	9.9
21	151117_ QC_A_3	Quality Control 3	1.721	591831	33752	15-Nov-17	21:29:37	12417.033	4.3
22	151117_ QC_B_3	Quality Control 3	1.727	3567002	203504	15-Nov-17	21:33:04	12984.740	11.8
23	151117_ QC_C_3	Quality Control 3	1.726	4683895	266958	15-Nov-17	21:36:32	12252.269	6.0

Cuantificación de Antiretrovirales en Leche Materna

Dataset: C:\MassLynx\MAHM_13Jul15.PRO\LINEALITY_ANALYSIS_11302017.qld

Last Altered: Thursday, November 30, 2017 15:04:17 Central Standard Time (Mexico)
Printed: Thursday, November 30, 2017 15:07:55 Central Standard Time (Mexico)

Compound name: RTV
Correlation coefficient: r = 0.998841, r^2 = 0.997683
Calibration curve: 0.00194096 * x + 0.0090028
Response type: Internal Std (Ref 5), Area * (IS Conc. / IS Area)
Curve type: Linear, Origin: Exclude, Weighting: 1/x, Axis trans: None

	Name	Sample Text	RT	Height	Area	Acq.Date	Acq.Time	IS Area	%Dev
1	151117_STD_1	Lower Limit of Q...	1.674	3859	225	15-Nov-17	18:11:21	13203.555	-16.9
2	151117_STD_2	Standard 2	1.671	6268	367	15-Nov-17	18:14:50	12271.620	7.5
3	151117_STD_3	Standard 3	1.674	10300	601	15-Nov-17	18:18:18	11723.644	9.0
4	151117_STD_4	Standard 4	1.672	27402	1577	15-Nov-17	18:21:48	13359.588	12.4
5	151117_STD_5	Standard 5	1.677	43390	2533	15-Nov-17	18:25:13	12602.851	-1.1
6	151117_STD_6	Standard 6	1.672	86656	4997	15-Nov-17	18:28:39	11994.957	5.0
7	151117_STD_7	Upper Limit of Q...	1.671	122562	7094	15-Nov-17	18:32:05	12430.945	-3.5
8	151117_Blank_22	Blank	1.680	535	35	15-Nov-17	18:50:33	38.652	
9	151117_ QC_A_1	Quality Control 1	1.672	13227	774	15-Nov-17	18:53:59	12167.331	-6.2
10	151117_ QC_B_1	Quality Control 1	1.674	79438	4577	15-Nov-17	18:57:25	12087.120	12.0
11	151117_ QC_C_1	Quality Control 1	1.674	105462	6070	15-Nov-17	19:00:53	11556.002	6.4
12	151117_S_1	SAMPLE	1.672	123663	7148	15-Nov-17	19:07:48	10489.766	
13	151117_S_2	SAMPLE	1.677	110752	6360	15-Nov-17	19:11:13	8039.864	
14	151117_S_3	SAMPLE	1.672	2632	159	15-Nov-17	19:14:39	11879.370	
15	151117_S_4	SAMPLE	1.674	16456	951	15-Nov-17	19:18:06	4767.146	
16	151117_S_5	SAMPLE	1.672	68427	3917	15-Nov-17	19:21:33	7595.172	
17	151117_S_6	SAMPLE	1.672	31519	1808	15-Nov-17	19:25:00	7552.594	
18	151117_ QC_A_2	Quality Control 2	1.672	15900	920	15-Nov-17	19:31:54	12742.463	8.5
19	151117_ QC_B_2	Quality Control 2	1.672	81382	4678	15-Nov-17	19:35:19	11741.281	18.0
20	151117_ QC_C_2	Quality Control 2	1.672	106972	6177	15-Nov-17	19:38:47	11770.134	6.3
21	151117_ QC_A_3	Quality Control 3	1.672	15050	863	15-Nov-17	21:29:37	12417.033	4.0
22	151117_ QC_B_3	Quality Control 3	1.672	78449	4505	15-Nov-17	21:33:04	12984.740	2.4
23	151117_ QC_C_3	Quality Control 3	1.671	106880	6123	15-Nov-17	21:36:32	12252.269	1.1

Cuantificación de Antiretrovirales en Leche Materna

Dataset: C:\MassLynx\MAHM_13Jul15.PRO\LINEALITY_ANALYSIS_11302017.qld

Last Altered: Thursday, November 30, 2017 15:04:17 Central Standard Time (Mexico)
Printed: Thursday, November 30, 2017 15:07:55 Central Standard Time (Mexico)

Compound name: SMV
Response Factor: 12512.5
RRF SD: 599.736, Relative SD: 4.79311
Response type: External Std, Area
Curve type: RF

	Name	Sample Text	RT	Height	Area	Acq.Date	Acq.Time	IS Area	%Dev
1	151117_STD_1	Lower Limit of Q...	2.129	228504	13204	15-Nov-17	18:11:21		5.5
2	151117_STD_2	Standard 2	2.122	211949	12272	15-Nov-17	18:14:50		-1.9
3	151117_STD_3	Standard 3	2.130	202553	11724	15-Nov-17	18:18:18		-6.3
4	151117_STD_4	Standard 4	2.122	230847	13360	15-Nov-17	18:21:48		6.8
5	151117_STD_5	Standard 5	2.128	216810	12603	15-Nov-17	18:25:13		0.7
6	151117_STD_6	Standard 6	2.128	206772	11995	15-Nov-17	18:28:39		-4.1
7	151117_STD_7	Upper Limit of Q...	2.127	214285	12431	15-Nov-17	18:32:05		-0.7
8	151117_Blank_22	Blank	2.136	518	39	15-Nov-17	18:50:33		-99.7
9	151117_QC_A_1	Quality Control 1	2.122	210101	12167	15-Nov-17	18:53:59		-2.8
10	151117_QC_B_1	Quality Control 1	2.130	208404	12087	15-Nov-17	18:57:25		-3.4
11	151117_QC_C_1	Quality Control 1	2.130	199365	11556	15-Nov-17	19:00:53		-7.6
12	151117_S_1	SAMPLE	2.127	181750	10490	15-Nov-17	19:07:48		-16.2
13	151117_S_2	SAMPLE	2.128	139461	8040	15-Nov-17	19:11:13		-35.7
14	151117_S_3	SAMPLE	2.128	205206	11879	15-Nov-17	19:14:39		-5.1
15	151117_S_4	SAMPLE	2.124	82708	4767	15-Nov-17	19:18:06		-61.9
16	151117_S_5	SAMPLE	2.128	131777	7595	15-Nov-17	19:21:33		-39.3
17	151117_S_6	SAMPLE	2.122	130417	7553	15-Nov-17	19:25:00		-39.6
18	151117_QC_A_2	Quality Control 2	2.128	221249	12742	15-Nov-17	19:31:54		1.8
19	151117_QC_B_2	Quality Control 2	2.128	203471	11741	15-Nov-17	19:35:19		-6.2
20	151117_QC_C_2	Quality Control 2	2.127	203667	11770	15-Nov-17	19:38:47		-5.9
21	151117_QC_A_3	Quality Control 3	2.122	215841	12417	15-Nov-17	21:29:37		-0.8
22	151117_QC_B_3	Quality Control 3	2.128	226920	12985	15-Nov-17	21:33:04		3.8
23	151117_QC_C_3	Quality Control 3	2.127	213530	12252	15-Nov-17	21:36:32		-2.1

Cuantificación de Antiretrovirales en Leche Materna

Dataset: C:\MassLynx\MAHM_13Jul15.PRO\LINEALITY_ANALYSIS_11302017.qld

Last Altered: Thursday, November 30, 2017 15:04:17 Central Standard Time (Mexico)
Printed: Thursday, November 30, 2017 15:07:55 Central Standard Time (Mexico)

Method: C:\MassLynx\MAHM_13Jul15.PRO\MethDB\071117_ZFG_NUEVO.mdb 30 Nov 2017 14:56:50
Calibration: 30 Nov 2017 15:04:17

Compound name: LMV
Correlation coefficient: r = 0.999074, r^2 = 0.998148
Calibration curve: 0.0012278 * x + 0.0217883
Response type: Internal Std (Ref 5), Area * (IS Conc. / IS Area)
Curve type: Linear, Origin: Exclude, Weighting: 1/x, Axis trans: None

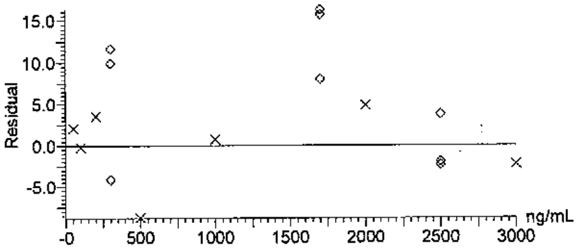

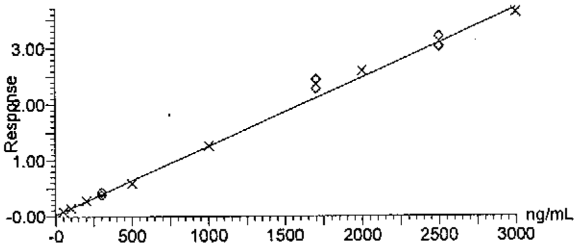

Compound name: ZDV
Correlation coefficient: r = 0.999649, r^2 = 0.999298
Calibration curve: 0.000901537 * x + 0.00453434
Response type: Internal Std (Ref 5), Area * (IS Conc. / IS Area)
Curve type: Linear, Origin: Exclude, Weighting: 1/x, Axis trans: None

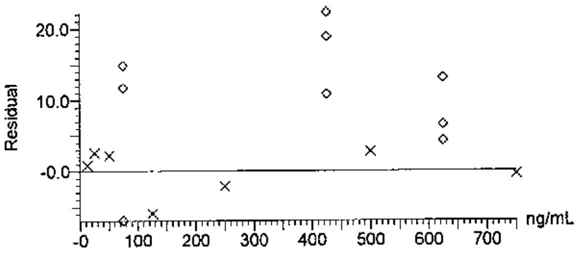

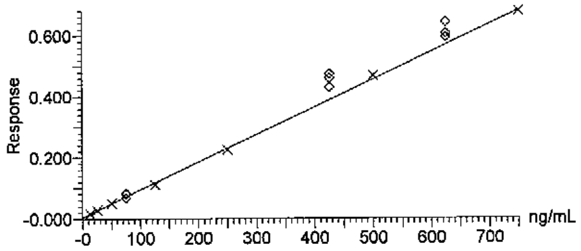

Compound name: LPV
Correlation coefficient: r = 0.998813, r^2 = 0.997627
Calibration curve: 0.00408171 * x + 0.163825
Response type: Internal Std (Ref 5), Area * (IS Conc. / IS Area)
Curve type: Linear, Origin: Exclude, Weighting: 1/x, Axis trans: None

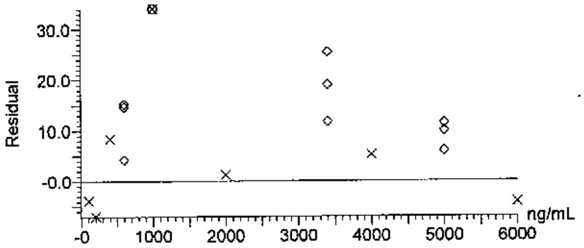

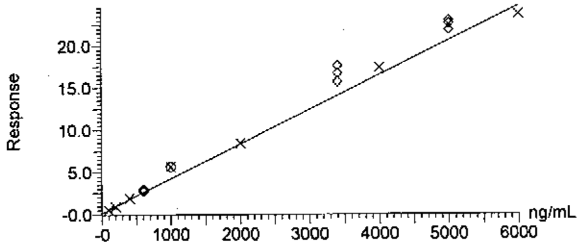

Cuantificación de Antiretrovirales en Leche Materna

Dataset: C:\MassLynx\MAHM_13Jul15.PRO\LINEALITY_ANALYSIS_11302017.qld

Last Altered: Thursday, November 30, 2017 15:04:17 Central Standard Time (Mexico)
Printed: Thursday, November 30, 2017 15:07:55 Central Standard Time (Mexico)

Compound name: RTV
Correlation coefficient: r = 0.998841, r^2 = 0.997683
Calibration curve: 0.00194096 * x + 0.0090028
Response type: Internal Std (Ref 5), Area * (IS Conc. / IS Area)
Curve type: Linear, Origin: Exclude, Weighting: 1/x, Axis trans: None

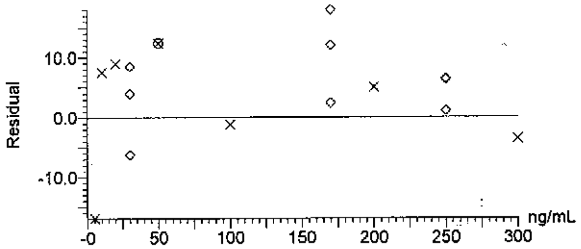

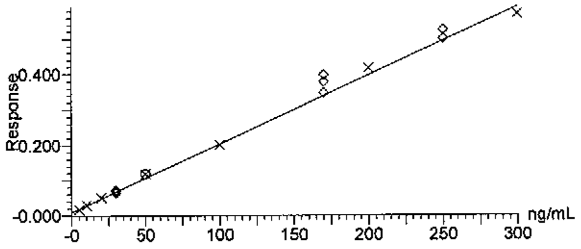

Compound name: SMV
Response Factor: 12512.5
RRF SD: 599.736, % Relative SD: 4.79311
Response type: External Std, Area
Curve type: RF

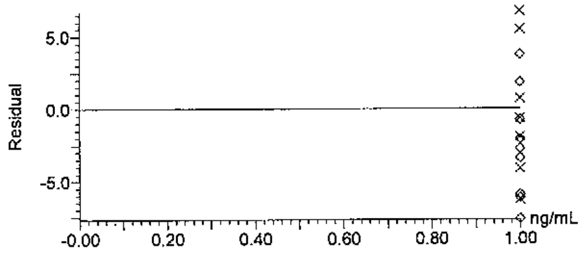

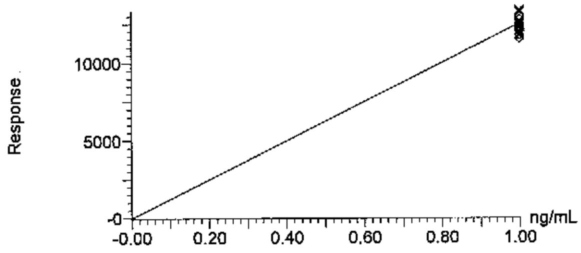

Quantify Compound Report MassLynx 4.1 SCN 843
Cuantificación de Antiretrovirales en Leche Materna

Dataset: C:\MassLynx\MAHM_13Jul15.PRO\LINEALITY_ANALYSIS_11302017.qld

Last Altered: Thursday, November 30, 2017 15:04:17 Central Standard Time (Mexico)
Printed: Thursday, November 30, 2017 15:07:55 Central Standard Time (Mexico)

Method: C:\MassLynx\MAHM_13Jul15.PRO\MethDB\071117_ZFG_NUEVO.mdb 30 Nov 2017 14:56:50
Calibration: 30 Nov 2017 15:04:17

Sample Name: 151117_STD_1

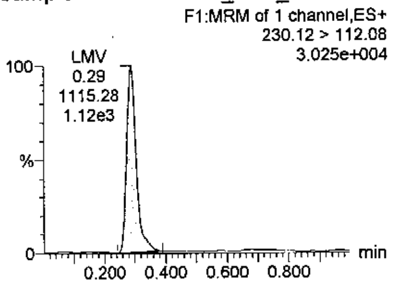

Sample Name: 151117_STD_2

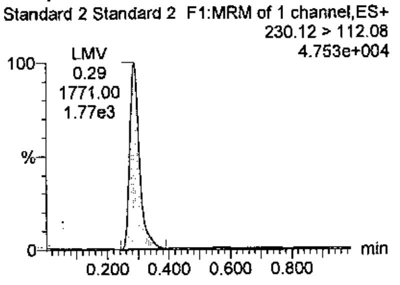

Sample Name: 151117_STD_3

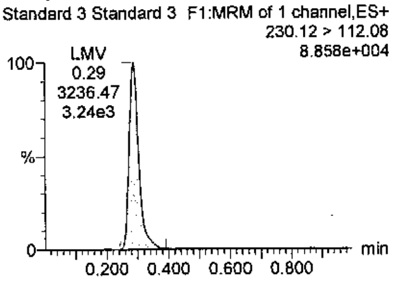

Sample Name: 151117_STD_4

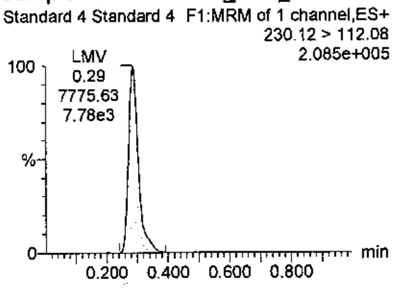

Sample Name: 151117_STD_5

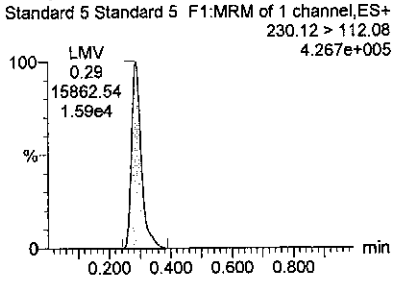

Sample Name: 151117_STD_6

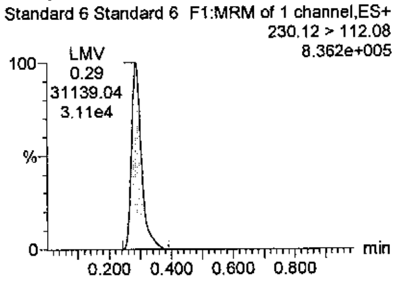

Sample Name: 151117_STD_7

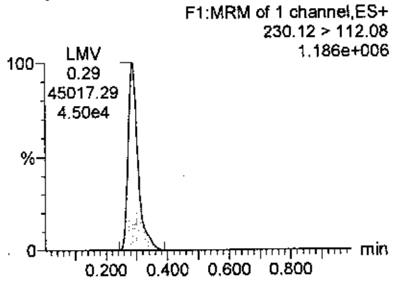

Sample Name: 151117_Blank_22

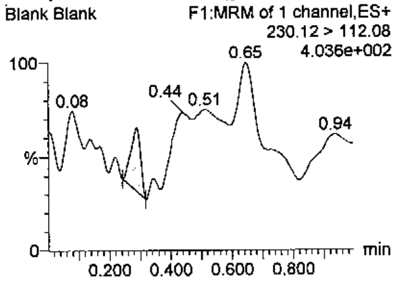

Sample Name: 151117_ QC_A_1

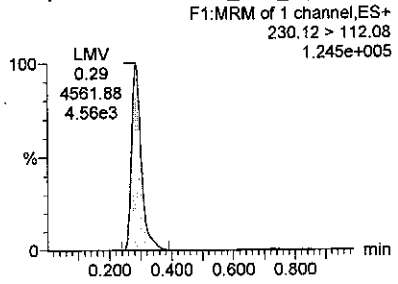

Sample Name: 151117_ QC_B_1

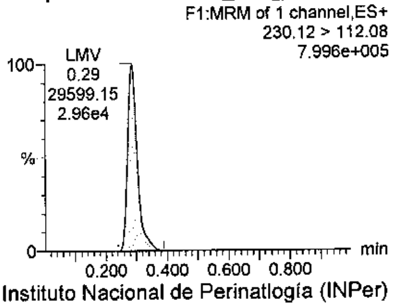

Sample Name: 151117_ QC_C_1

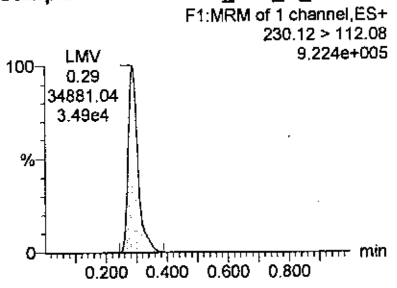

Sample Name: 151117_S_1

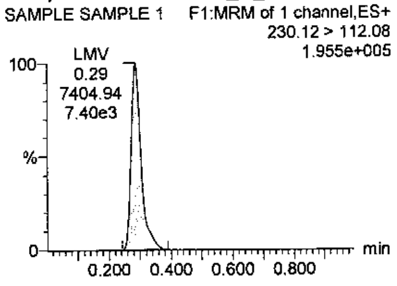

Dataset: C:\MassLynx\MAHM_13Jul15.PRO\LINEALITY_ANALYSIS_11302017.qld

Last Altered: Thursday, November 30, 2017 15:04:17 Central Standard Time (Mexico)
Printed: Thursday, November 30, 2017 15:07:55 Central Standard Time (Mexico)

Sample Name: 151117_S_2
SAMPLE SAMPLE 2 F1:MRM of 1 channel,ES+
230.12 > 112.08

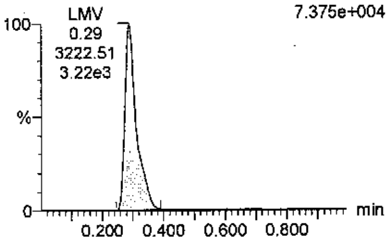

Sample Name: 151117_S_3
SAMPLE SAMPLE 3 F1:MRM of 1 channel,ES+
230.12 > 112.08

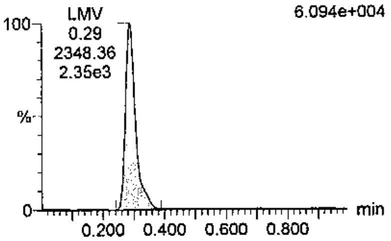

Sample Name: 151117_S_4
SAMPLE SAMPLE 4 F1:MRM of 1 channel,ES+
230.12 > 112.08

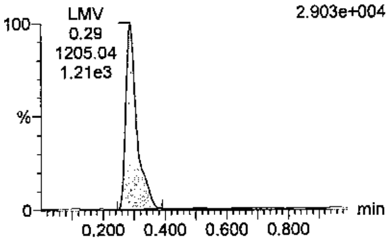

Sample Name: 151117_S_5
SAMPLE SAMPLE 5 F1:MRM of 1 channel,ES+
230.12 > 112.08

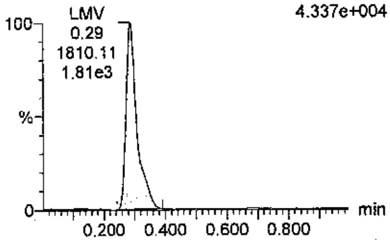

Sample Name: 151117_S_6
SAMPLE SAMPLE 6 F1:MRM of 1 channel,ES+
230.12 > 112.08

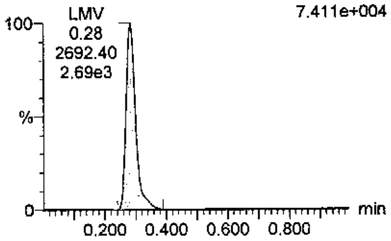

Sample Name: 151117_ QC_A_2
F1:MRM of 1 channel,ES+
230.12 > 112.08

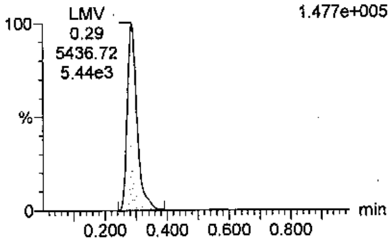

Sample Name: 151117_ QC_B_2
F1:MRM of 1 channel,ES+
230.12 > 112.08

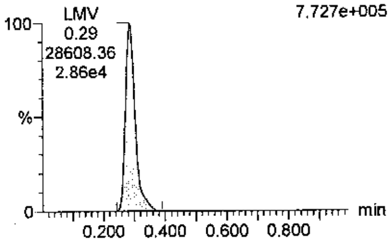

Sample Name: 151117_ QC_C_2
F1:MRM of 1 channel,ES+
230.12 > 112.08

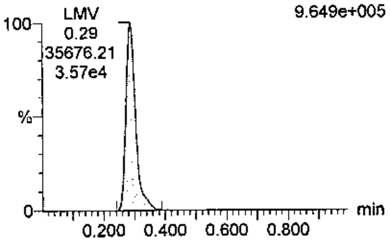

Sample Name: 151117_ QC_A_3
F1:MRM of 1 channel,ES+
230.12 > 112.08

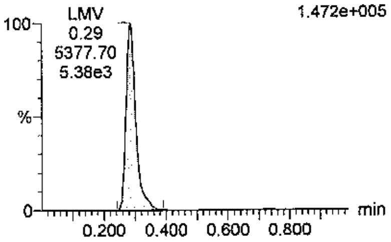

Sample Name: 151117_ QC_B_3
F1:MRM of 1 channel,ES+
230.12 > 112.08

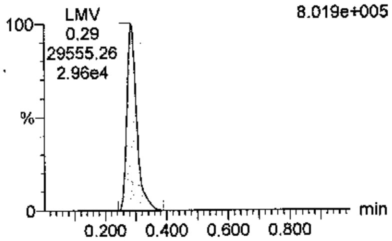

Sample Name: 151117_ QC_C_3
F1:MRM of 1 channel,ES+
230.12 > 112.08

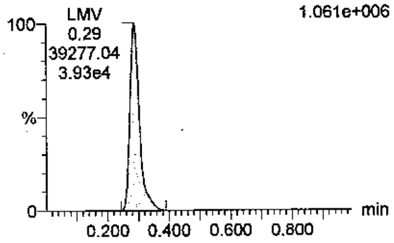

Dataset: C:\MassLynx\MAHM_13Jul15.PRO\LINEALITY_ANALYSIS_11302017.qld

Last Altered: Thursday, November 30, 2017 15:04:17 Central Standard Time (Mexico)
Printed: Thursday, November 30, 2017 15:07:55 Central Standard Time (Mexico)

	#	Name	Type	Std. Conc	RT	Area	IS Area	Response	Primar...	ng/mL	%Dev
1	1	151117_STD_1	Standard	50.000	0.29	1115.284	13203.555	0.084	bb	51.1	2.1
2	2	151117_STD_2	Standard	100.000	0.29	1770.996	12271.620	0.144	bb	99.8	-0.2
3	3	151117_STD_3	Standard	200.000	0.29	3236.470	11723.644	0.276	bb	207.1	3.5
4	4	151117_STD_4	Standard	500.000	0.29	7775.626	13359.588	0.582	bb	456.3	-8.7
5	5	151117_STD_5	Standard	1000.000	0.29	15862.536	12602.851	1.259	bb	1007.4	0.7
6	6	151117_STD_6	Standard	2000.000	0.29	31139.037	11994.957	2.596	bb	2096.6	4.8
7	7	151117_STD_7	Standard	3000.000	0.29	45017.285	12430.945	3.621	bb	2931.8	-2.3
8	8	151117_Blank_22	Blank		0.29	5.174	38.652	0.134	bb	91.3	
9	9	151117_ QC_A_1	QC	300.000	0.29	4561.879	12167.331	0.375	bb	287.6	-4.1
10	10	151117_ QC_B_1	QC	1700.000	0.29	29599.146	12087.120	2.449	bb	1976.7	16.3
11	11	151117_ QC_C_1	QC	2500.000	0.29	34881.035	11556.002	3.018	bb	2440.7	-2.4
12	12	151117_S_1	Analyte		0.29	7404.940	10489.766	0.706	bb	557.2	
13	13	151117_S_2	Analyte		0.29	3222.515	8039.864	0.401	bb	308.7	
14	14	151117_S_3	Analyte		0.29	2348.365	11879.370	0.198	bb	143.3	
15	15	151117_S_4	Analyte		0.29	1205.043	4767.146	0.253	bb	188.1	
16	16	151117_S_5	Analyte		0.29	1810.110	7595.172	0.238	bb	176.4	
17	17	151117_S_6	Analyte		0.28	2692.400	7552.594	0.356	bb	272.6	
18	18	151117_ QC_A_2	QC	300.000	0.29	5436.715	12742.463	0.427	bb	329.8	9.9
19	19	151117_ QC_B_2	QC	1700.000	0.29	28608.363	11741.281	2.437	bb	1966.8	15.7
20	20	151117_ QC_C_2	QC	2500.000	0.29	35676.215	11770.134	3.031	bb	2451.0	-2.0
21	21	151117_ QC_A_3	QC	300.000	0.29	5377.701	12417.033	0.433	bb	335.0	11.7
22	22	151117_ QC_B_3	QC	1700.000	0.29	29555.264	12984.740	2.276	bb	1836.1	8.0
23	23	151117_ QC_C_3	QC	2500.000	0.29	39277.035	12252.269	3.206	bb	2593.2	3.7

Dataset: C:\MassLynx\MAHM_13Jul15.PRO\LINEALITY_ANALYSIS_11302017.qld

Last Altered: Thursday, November 30, 2017 15:04:17 Central Standard Time (Mexico)
Printed: Thursday, November 30, 2017 15:07:55 Central Standard Time (Mexico)

Sample Name: 151117_STD_1
F2:MRM of 1 channel,ES+
268.2 > 127.1
3.582e+003

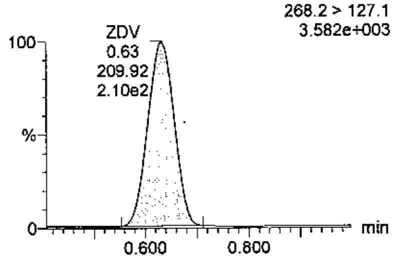

Sample Name: 151117_STD_2
Standard 2 Standard 2 F2:MRM of 1 channel,ES+
268.2 > 127.1
5.887e+003

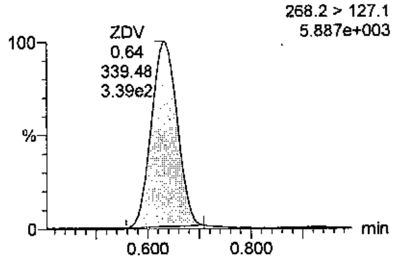

Sample Name: 151117_STD_3
Standard 3 Standard 3 F2:MRM of 1 channel,ES+
268.2 > 127.1
9.974e+003

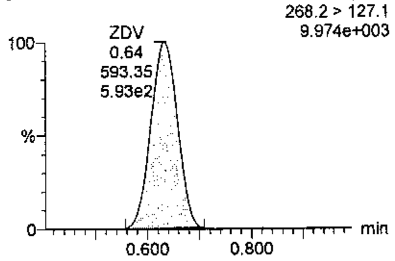

Sample Name: 151117_STD_4
Standard 4 Standard 4 F2:MRM of 1 channel,ES+
268.2 > 127.1
2.519e+004

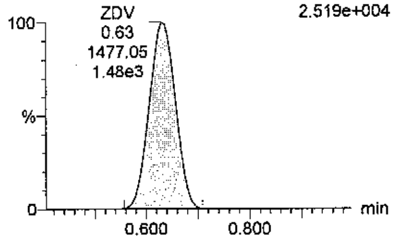

Sample Name: 151117_STD_5
Standard 5 Standard 5 F2:MRM of 1 channel,ES+
268.2 > 127.1
4.861e+004

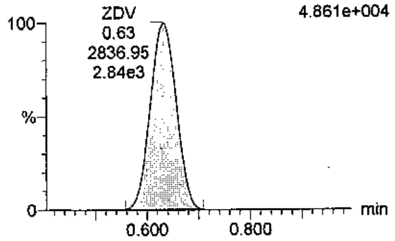

Sample Name: 151117_STD_6
Standard 6 Standard 6 F2:MRM of 1 channel,ES+
268.2 > 127.1
9.608e+004

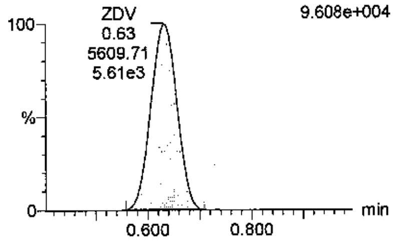

Sample Name: 151117_STD_7
F2:MRM of 1 channel,ES+
268.2 > 127.1
1.458e+005

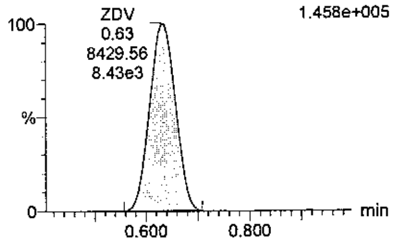

Sample Name: 151117_Blank_22
Blank Blank F2:MRM of 1 channel,ES+
268.2 > 127.1
1.010e+002

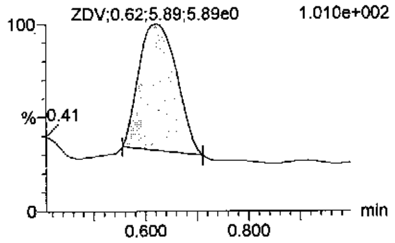

Sample Name: 151117_ QC_A_1
F2:MRM of 1 channel,ES+
268.2 > 127.1
1.413e+004

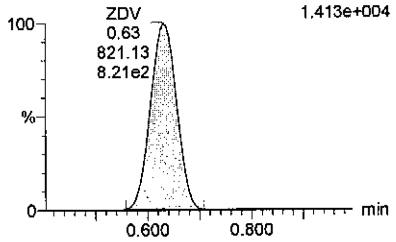

Sample Name: 151117_ QC_B_1
F2:MRM of 1 channel,ES+
268.2 > 127.1
9.766e+004

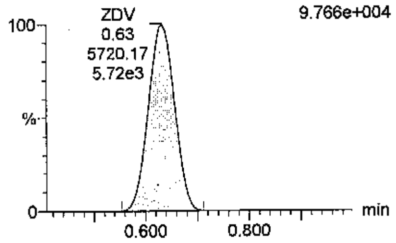

Sample Name: 151117_ QC_C_1
F2:MRM of 1 channel,ES+
268.2 > 127.1
1.285e+005

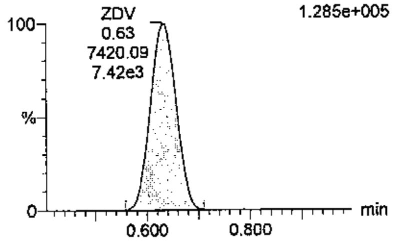

Sample Name: 151117_S_1
SAMPLE SAMPLE 1 F2:MRM of 1 channel,ES+
268.2 > 127.1
7.602e+003

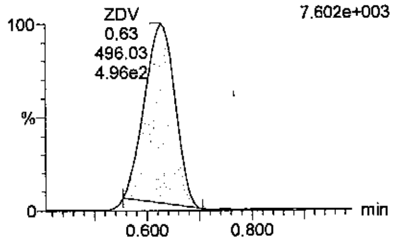

Dataset: C:\MassLynx\MAHM_13Jul15.PRO\LINEALITY_ANALYSIS_11302017.qld

Last Altered: Thursday, November 30, 2017 15:04:17 Central Standard Time (Mexico)
Printed: Thursday, November 30, 2017 15:07:55 Central Standard Time (Mexico)

Sample Name: 151117_S_2
SAMPLE SAMPLE 2 F2:MRM of 1 channel,ES+
268.2 > 127.1
2.501e+003

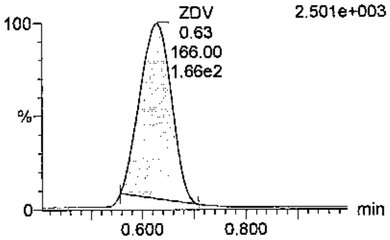

Sample Name: 151117_S_3
SAMPLE SAMPLE 3 F2:MRM of 1 channel,ES+
268.2 > 127.1
1.910e+004

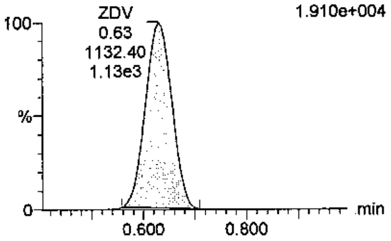

Sample Name: 151117_S_4
SAMPLE SAMPLE 4 F2:MRM of 1 channel,ES+
268.2 > 127.1
6.532e+003

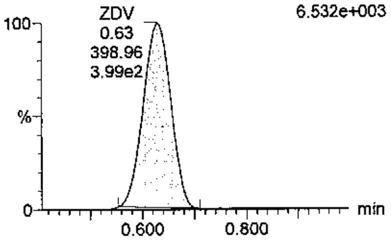

Sample Name: 151117_S_5
SAMPLE SAMPLE 5 F2:MRM of 1 channel,ES+
268.2 > 127.1
2.682e+003

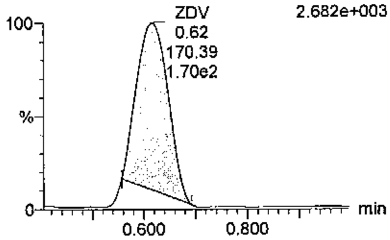

Sample Name: 151117_S_6
SAMPLE SAMPLE 6 F2:MRM of 1 channel,ES+
268.2 > 127.1
5.142e+004

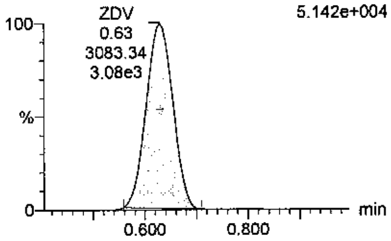

Sample Name: 151117_ QC_A_2
F2:MRM of 1 channel,ES+
268.2 > 127.1
1.770e+004

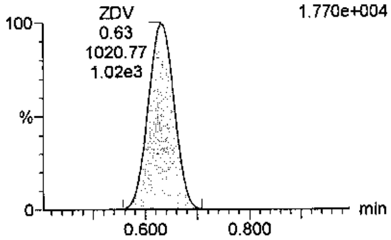

Sample Name: 151117_ QC_B_2
F2:MRM of 1 channel,ES+
268.2 > 127.1
9.261e+004

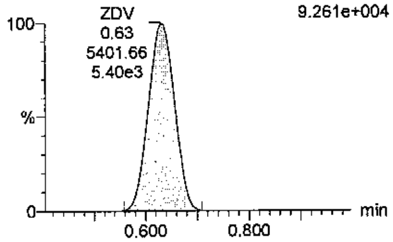

Sample Name: 151117_ QC_C_2
F2:MRM of 1 channel,ES+
268.2 > 127.1
1.187e+005

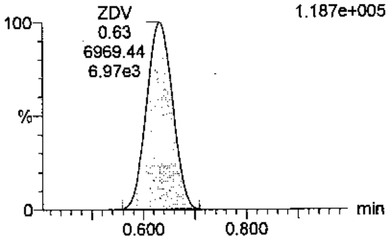

Sample Name: 151117_ QC_A_3
F2:MRM of 1 channel,ES+
268.2 > 127.1
1.751e+004

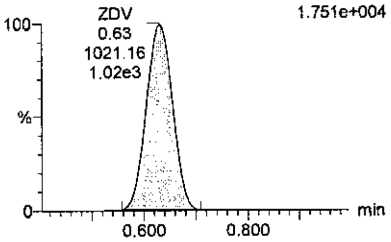

Sample Name: 151117_ QC_B_3
F2:MRM of 1 channel,ES+
268.2 > 127.1
9.587e+004

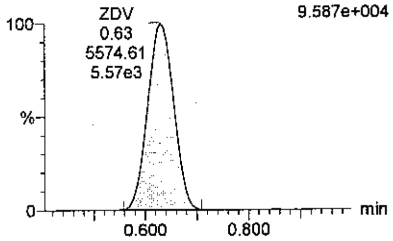

Sample Name: 151117_ QC_C_3
F2:MRM of 1 channel,ES+
268.2 > 127.1
1.290e+005

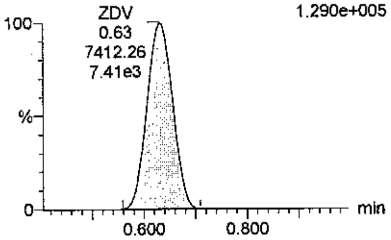

Dataset: C:\MassLynx\MAHM_13Jul15.PRO\LINEALITY_ANALYSIS_11302017.qld

Last Altered: Thursday, November 30, 2017 15:04:17 Central Standard Time (Mexico)
Printed: Thursday, November 30, 2017 15:07:55 Central Standard Time (Mexico)

	#	Name	Type	Std. Conc	RT	Area	IS Area	Response	Primar...	ng/mL	%Dev
1	1	151117_STD_1	Standard	12.500	0.63	209.922	13203.555	0.016	bb	12.6	0.8
2	2	151117_STD_2	Standard	25.000	0.64	339.482	12271.620	0.028	bb	25.7	2.6
3	3	151117_STD_3	Standard	50.000	0.64	593.346	11723.644	0.051	bb	51.1	2.2
4	4	151117_STD_4	Standard	125.000	0.63	1477.054	13359.588	0.111	bb	117.6	-5.9
5	5	151117_STD_5	Standard	250.000	0.63	2836.954	12602.851	0.225	bb	244.7	-2.1
6	6	151117_STD_6	Standard	500.000	0.63	5609.708	11994.957	0.468	bb	513.7	2.7
7	7	151117_STD_7	Standard	750.000	0.63	8429.557	12430.945	0.678	bb	747.1	-0.4
8	8	151117_Blank_22	Blank		0.62	5.893	38.652	0.152	bb	164.1	
9	9	151117_ QC_A_1	QC	75.000	0.63	821.127	12167.331	0.067	bb	69.8	-6.9
10	10	151117_ QC_B_1	QC	425.000	0.63	5720.167	12087.120	0.473	bb	519.9	22.3
11	11	151117_ QC_C_1	QC	625.000	0.63	7420.089	11556.002	0.642	bb	707.2	13.2
12	12	151117_S_1	Analyte		0.63	496.033	10489.766	0.047	bb	47.4	
13	13	151117_S_2	Analyte		0.63	165.998	8039.864	0.021	bb	17.9	
14	14	151117_S_3	Analyte		0.63	1132.402	11879.370	0.095	bb	100.7	
15	15	151117_S_4	Analyte		0.63	398.958	4767.146	0.084	bb	87.8	
16	16	151117_S_5	Analyte		0.62	170.387	7595.172	0.022	bb	19.9	
17	17	151117_S_6	Analyte		0.63	3083.337	7552.594	0.408	bb	447.8	
18	18	151117_ QC_A_2	QC	75.000	0.63	1020.772	12742.463	0.080	bb	83.8	11.8
19	19	151117_ QC_B_2	QC	425.000	0.63	5401.661	11741.281	0.460	bb	505.3	18.9
20	20	151117_ QC_C_2	QC	625.000	0.63	6969.442	11770.134	0.592	bb	651.8	4.3
21	21	151117_ QC_A_3	QC	75.000	0.63	1021.158	12417.033	0.082	bb	86.2	14.9
22	22	151117_ QC_B_3	QC	425.000	0.63	5574.612	12984.740	0.429	bb	471.2	10.9
23	23	151117_ QC_C_3	QC	625.000	0.63	7412.258	12252.269	0.605	bb	666.0	6.6

Cuantificación de Antiretrovirales en Leche Materna

Dataset: C:\MassLynx\MAHM_13Jul15.PRO\LINEALITY_ANALYSIS_11302017.qld

Last Altered: Thursday, November 30, 2017 15:04:17 Central Standard Time (Mexico)
Printed: Thursday, November 30, 2017 15:07:55 Central Standard Time (Mexico)

Sample Name: 151117_STD_1

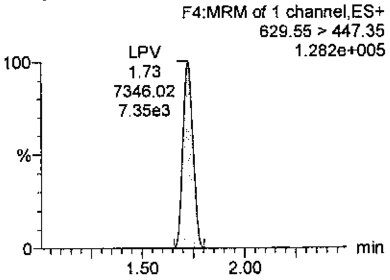

Sample Name: 151117_STD_2

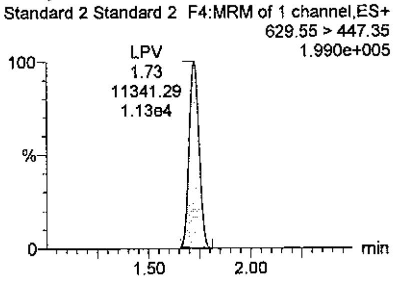

Sample Name: 151117_STD_3

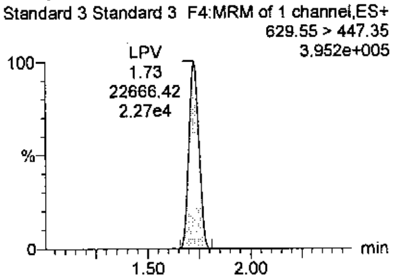

Sample Name: 151117_STD_4

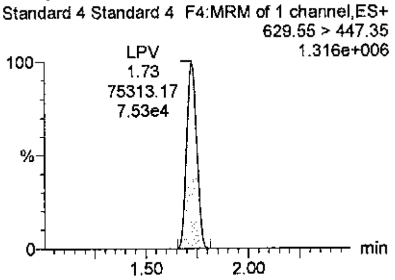

Sample Name: 151117_STD_5

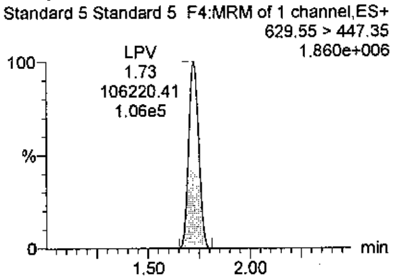

Sample Name: 151117_STD_6

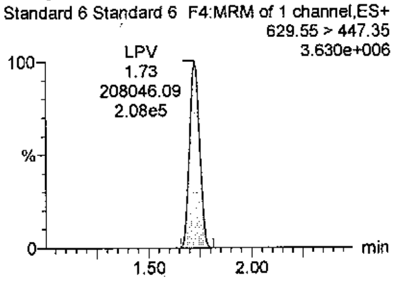

Sample Name: 151117_STD_7

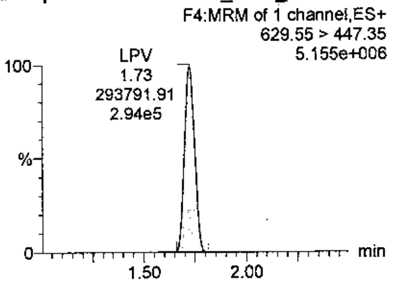

Sample Name: 151117_Blank_22

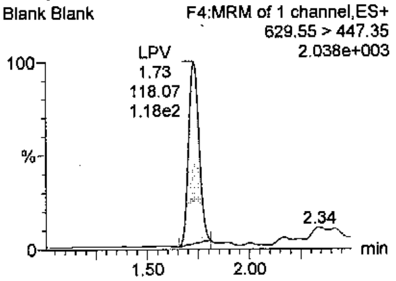

Sample Name: 151117_ QC_A_1

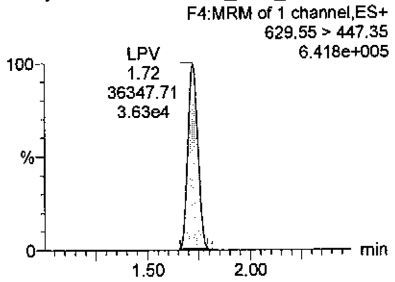

Sample Name: 151117_ QC_B_1

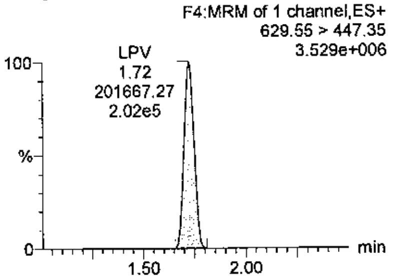

Sample Name: 151117_ QC_C_1

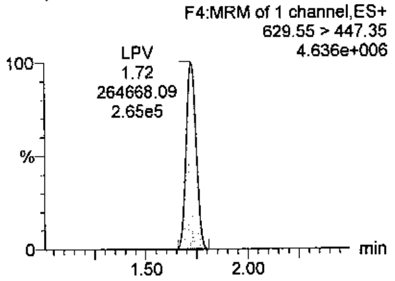

Sample Name: 151117_S_1

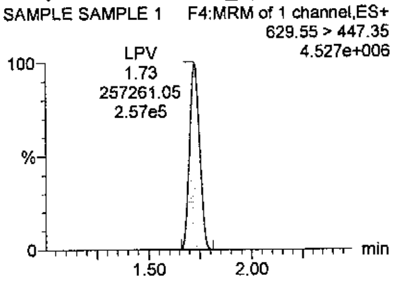

Sample Name: 151117_S_2
SAMPLE SAMPLE 2 F4:MRM of 1 channel,ES+
629.55 > 447.35
2.772e+006

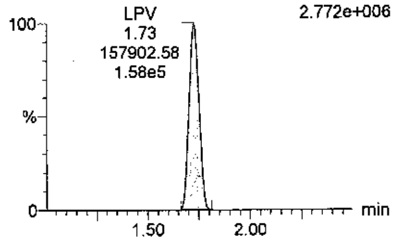

Sample Name: 151117_S_3
SAMPLE SAMPLE 3 F4:MRM of 1 channel,ES+
629.55 > 447.35
7.236e+004

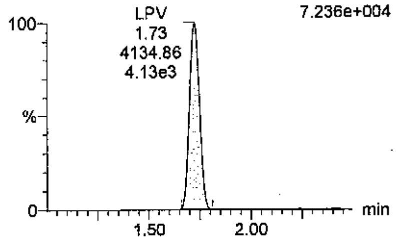

Sample Name: 151117_S_4
SAMPLE SAMPLE 4 F4:MRM of 1 channel,ES+
629.55 > 447.35
8.737e+005

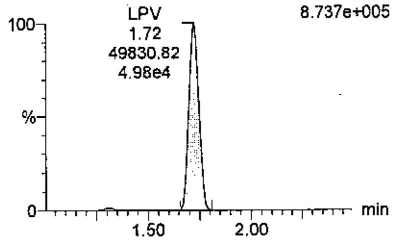

Sample Name: 151117_S_5
SAMPLE SAMPLE 5 F4:MRM of 1 channel,ES+
629.55 > 447.35
1.571e+006

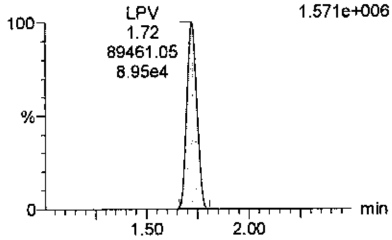

Sample Name: 151117_S_6
SAMPLE SAMPLE 6 F4:MRM of 1 channel,ES+
629.55 > 447.35
6.870e+005

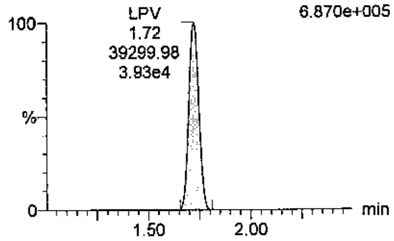

Sample Name: 151117_ QC_A_2
F4:MRM of 1 channel,ES+
629.55 > 447.35
6.672e+005

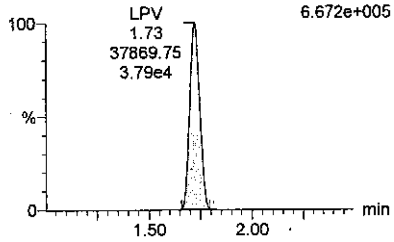

Sample Name: 151117_ QC_B_2
F4:MRM of 1 channel,ES+
629.55 > 447.35
3.630e+006

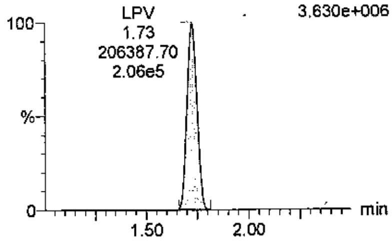

Sample Name: 151117_ QC_C_2
F4:MRM of 1 channel,ES+
629.55 > 447.35
4.669e+006

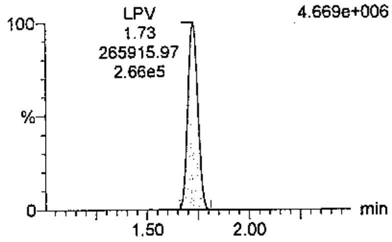

Sample Name: 151117_ QC_A_3
F4:MRM of 1 channel,ES+
629.55 > 447.35
5.924e+005

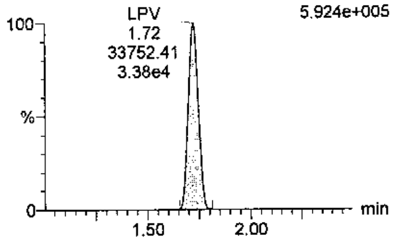

Sample Name: 151117_ QC_B_3
F4:MRM of 1 channel,ES+
629.55 > 447.35
3.569e+006

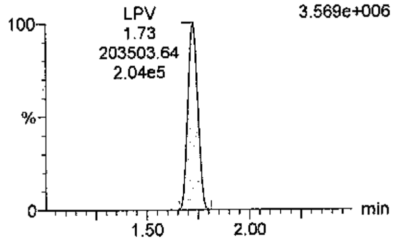

Sample Name: 151117_ QC_C_3
F4:MRM of 1 channel,ES+
629.55 > 447.35
4.696e+006

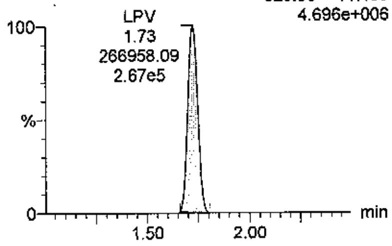

Dataset: C:\MassLynx\MAHM_13Jul15.PRO\LINEALITY_ANALYSIS_11302017.qld

Last Altered: Thursday, November 30, 2017 15:04:17 Central Standard Time (Mexico)
Printed: Thursday, November 30, 2017 15:07:55 Central Standard Time (Mexico)

	#	Name	Type	Std. Conc	RT	Area	IS Area	Response	Primar...	ng/mL	%Dev
1	1	151117_STD_1	Standard	100.000	1.73	7346.025	13203.555	0.556	MM	96.2	-3.8
2	2	151117_STD_2	Standard	200.000	1.73	11341.294	12271.620	0.924	bb	186.3	-6.9
3	3	151117_STD_3	Standard	400.000	1.73	22666.422	11723.644	1.933	bb	433.5	8.4
4	4	151117_STD_4	Standard	1000.000	1.73	75313.172	13359.588	5.637	bbX	1341.0	34.1
5	5	151117_STD_5	Standard	2000.000	1.73	106220.406	12602.851	8.428	bb	2024.8	1.2
6	6	151117_STD_6	Standard	4000.000	1.73	208046.094	11994.957	17.344	bb	4209.2	5.2
7	7	151117_STD_7	Standard	6000.000	1.73	293791.906	12430.945	23.634	bb	5750.1	-4.2
8	8	151117_Blank_22	Blank		1.73	118.068	38.652	3.055	bb	708.2	
9	9	151117_ QC_A_1	QC	600.000	1.72	36347.711	12167.331	2.987	MM	691.7	15.3
10	10	151117_ QC_B_1	QC	3400.000	1.72	201667.266	12087.120	16.684	bb	4047.5	19.0
11	11	151117_ QC_C_1	QC	5000.000	1.72	264668.094	11556.002	22.903	bb	5571.0	11.4
12	12	151117_S_1	Analyte		1.73	257261.047	10489.766	24.525	bb	5968.4	
13	13	151117_S_2	Analyte		1.73	157902.578	8039.864	19.640	bb	4771.6	
14	14	151117_S_3	Analyte		1.73	4134.857	11879.370	0.348	bb	45.1	
15	15	151117_S_4	Analyte		1.72	49830.816	4767.146	10.453	bb	2520.8	
16	16	151117_S_5	Analyte		1.72	89461.047	7595.172	11.779	bb	2845.6	
17	17	151117_S_6	Analyte		1.72	39299.977	7552.594	5.204	bb	1234.7	
18	18	151117_ QC_A_2	QC	600.000	1.73	37869.750	12742.463	2.972	MM	688.0	14.7
19	19	151117_ QC_B_2	QC	3400.000	1.73	206387.703	11741.281	17.578	bb	4266.4	25.5
20	20	151117_ QC_C_2	QC	5000.000	1.73	265915.969	11770.134	22.592	bb	5494.9	9.9
21	21	151117_ QC_A_3	QC	600.000	1.72	33752.406	12417.033	2.718	bb	625.8	4.3
22	22	151117_ QC_B_3	QC	3400.000	1.73	203503.641	12984.740	15.673	bb	3799.6	11.8
23	23	151117_ QC_C_3	QC	5000.000	1.73	266958.094	12252.269	21.788	bb	5297.9	6.0

Dataset: C:\MassLynx\MAHM_13Jul15.PRO\LINEALITY_ANALYSIS_11302017.qld

Last Altered: Thursday, November 30, 2017 15:04:17 Central Standard Time (Mexico)
Printed: Thursday, November 30, 2017 15:07:55 Central Standard Time (Mexico)

Sample Name: 151117_STD_1
F5:MRM of 1 channel,ES+
721.5 > 296.2, 268.2, 171.10
3.923e+003

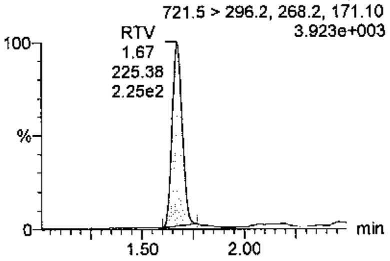

Sample Name: 151117_STD_2
Standard 2 Standard 2 F5:MRM of 1 channel,ES+
721.5 > 296.2, 268.2, 171.10
6.315e+003

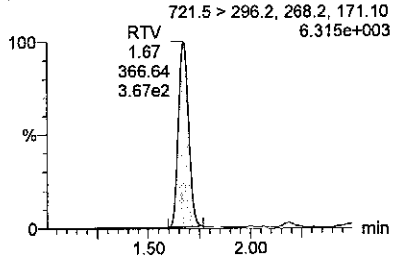

Sample Name: 151117_STD_3
Standard 3 Standard 3 F5:MRM of 1 channel,ES+
721.5 > 296.2, 268.2, 171.10
1.034e+004

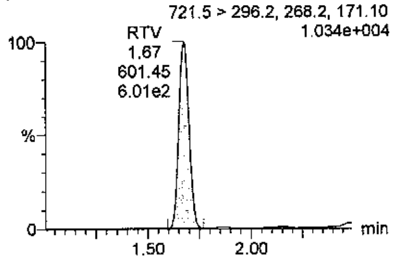

Sample Name: 151117_STD_4
Standard 4 Standard 4 F5:MRM of 1 channel,ES+
721.5 > 296.2, 268.2, 171.10
2.745e+004

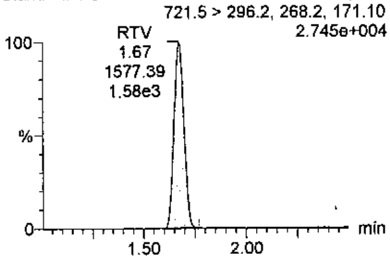

Sample Name: 151117_STD_5
Standard 5 Standard 5 F5:MRM of 1 channel,ES+
721.5 > 296.2, 268.2, 171.10
4.348e+004

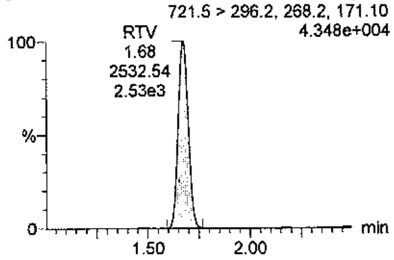

Sample Name: 151117_STD_6
Standard 6 Standard 6 F5:MRM of 1 channel,ES+
721.5 > 296.2, 268.2, 171.10
8.678e+004

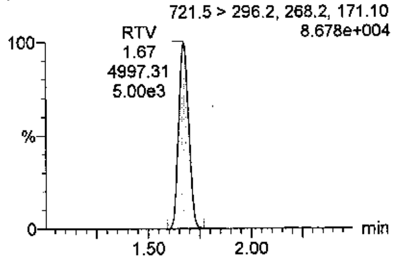

Sample Name: 151117_STD_7
F5:MRM of 1 channel,ES+
721.5 > 296.2, 268.2, 171.10
1.227e+005

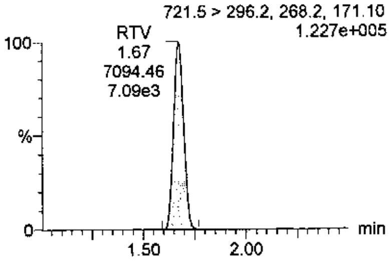

Sample Name: 151117_Blank_22
Blank Blank F5:MRM of 1 channel,ES+
721.5 > 296.2, 268.2, 171.10
5.678e+002

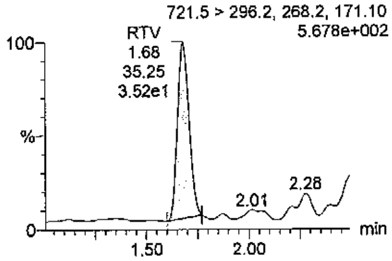

Sample Name: 151117_ QC_A_1
F5:MRM of 1 channel,ES+
721.5 > 296.2, 268.2, 171.10
1.328e+004

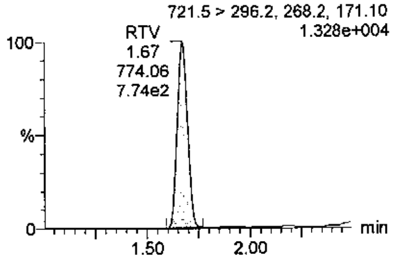

Sample Name: 151117_ QC_B_1
F5:MRM of 1 channel,ES+
721.5 > 296.2, 268.2, 171.10
7.956e+004

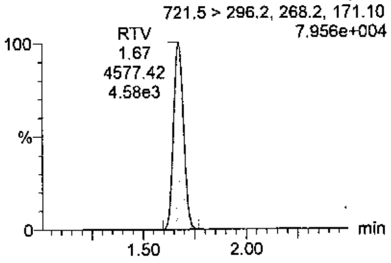

Sample Name: 151117_ QC_C_1
F5:MRM of 1 channel,ES+
721.5 > 296.2, 268.2, 171.10
1.056e+005

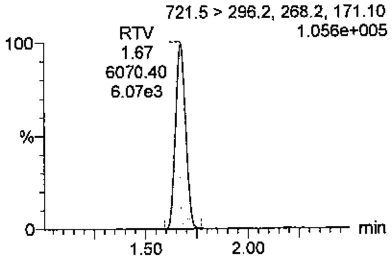

Sample Name: 151117_S_1
SAMPLE SAMPLE 1 F5:MRM of 1 channel,ES+
721.5 > 296.2, 268.2, 171.10
1.238e+005

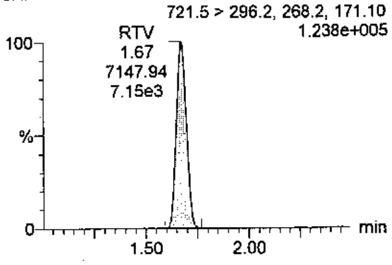

Cuantificación de Antiretrovirales en Leche Materna

Dataset: C:\MassLynx\MAHM_13Jul15.PRO\LINEALITY_ANALYSIS_11302017.qld

Last Altered: Thursday, November 30, 2017 15:04:17 Central Standard Time (Mexico)
Printed: Thursday, November 30, 2017 15:07:55 Central Standard Time (Mexico)

Sample Name: 151117_S_2
SAMPLE SAMPLE 2 F5:MRM of 1 channel,ES+
721.5 > 296.2, 268.2, 171.10

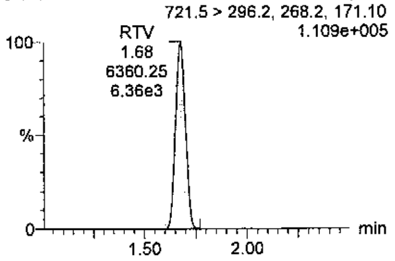

Sample Name: 151117_S_3
SAMPLE SAMPLE 3 F5:MRM of 1 channel,ES+
721.5 > 296.2, 268.2, 171.10

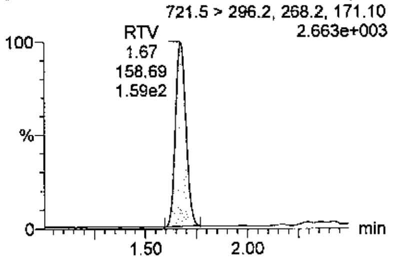

Sample Name: 151117_S_4
SAMPLE SAMPLE 4 F5:MRM of 1 channel,ES+
721.5 > 296.2, 268.2, 171.10

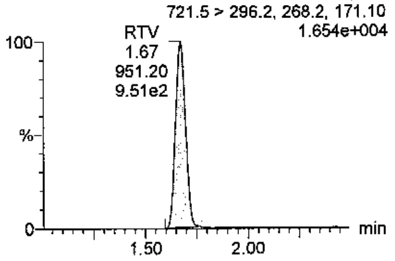

Sample Name: 151117_S_5
SAMPLE SAMPLE 5 F5:MRM of 1 channel,ES+
721.5 > 296.2, 268.2, 171.10

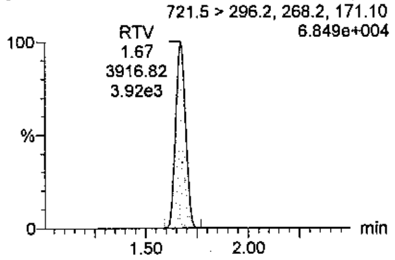

Sample Name: 151117_S_6
SAMPLE SAMPLE 6 F5:MRM of 1 channel,ES+
721.5 > 296.2, 268.2, 171.10

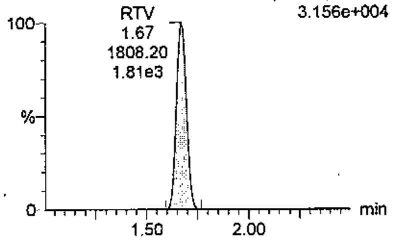

Sample Name: 151117_ QC_A_2
F5:MRM of 1 channel,ES+
721.5 > 296.2, 268.2, 171.10

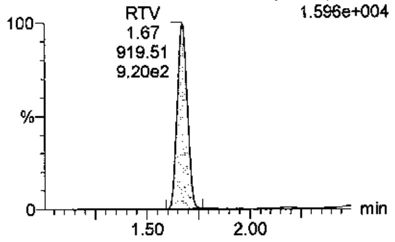

Sample Name: 151117_ QC_B_2
F5:MRM of 1 channel,ES+
721.5 > 296.2, 268.2, 171.10

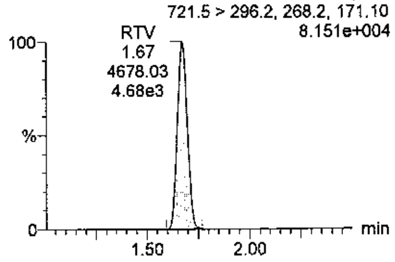

Sample Name: 151117_ QC_C_2
F5:MRM of 1 channel,ES+
721.5 > 296.2, 268.2, 171.10

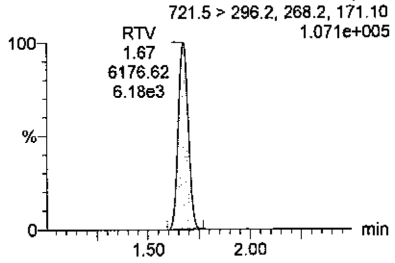

Sample Name: 151117_ QC_A_3
F5:MRM of 1 channel,ES+
721.5 > 296.2, 268.2, 171.10

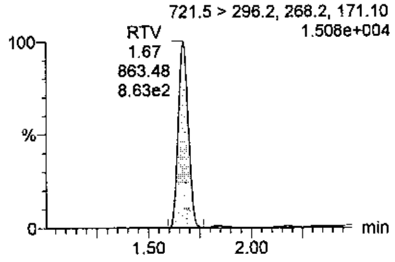

Sample Name: 151117_ QC_B_3
F5:MRM of 1 channel,ES+
721.5 > 296.2, 268.2, 171.10

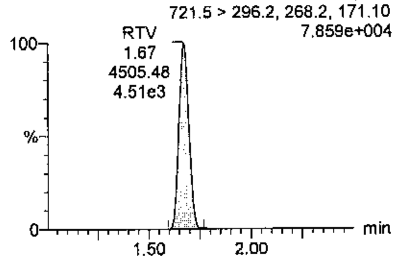

Sample Name: 151117_ QC_C_3
F5:MRM of 1 channel,ES+
721.5 > 296.2, 268.2, 171.10

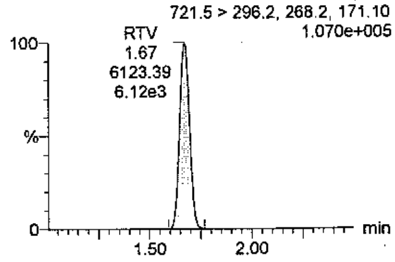

Dataset: C:\MassLynx\MAHM_13Jul15.PRO\LINEALITY_ANALYSIS_11302017.qld

Last Altered: Thursday, November 30, 2017 15:04:17 Central Standard Time (Mexico)
Printed: Thursday, November 30, 2017 15:07:55 Central Standard Time (Mexico)

	#	Name	Type	Std. Conc	RT	Area	IS Area	Response	Primar...	ng/mL	%Dev
1	1	151117_STD_1	Standard	5.000	1.67	225.384	13203.555	0.017	bb	4.2	-16.9
2	2	151117_STD_2	Standard	10.000	1.67	366.643	12271.620	0.030	bb	10.8	7.5
3	3	151117_STD_3	Standard	20.000	1.67	601.452	11723.644	0.051	bb	21.8	9.0
4	4	151117_STD_4	Standard	50.000	1.67	1577.387	13359.588	0.118	bbX	56.2	12.4
5	5	151117_STD_5	Standard	100.000	1.68	2532.539	12602.851	0.201	bb	98.9	-1.1
6	6	151117_STD_6	Standard	200.000	1.67	4997.311	11994.957	0.417	bb	210.0	5.0
7	7	151117_STD_7	Standard	300.000	1.67	7094.459	12430.945	0.571	bb	289.4	-3.5
8	8	151117_Blank_22	Blank		1.68	35.249	38.652	0.912	bb	465.2	
9	9	151117_ QC_A_1	QC	30.000	1.67	774.059	12167.331	0.064	bb	28.1	-6.2
10	10	151117_ QC_B_1	QC	170.000	1.67	4577.423	12087.120	0.379	bb	190.5	12.0
11	11	151117_ QC_C_1	QC	250.000	1.67	6070.399	11556.002	0.525	bb	266.0	6.4
12	12	151117_S_1	Analyte		1.67	7147.935	10489.766	0.681	bb	346.4	
13	13	151117_S_2	Analyte		1.68	6360.246	8039.864	0.791	bb	402.9	
14	14	151117_S_3	Analyte		1.67	158.687	11879.370	0.013	bb	2.2	
15	15	151117_S_4	Analyte		1.67	951.203	4767.146	0.200	bb	98.2	
16	16	151117_S_5	Analyte		1.67	3916.818	7595.172	0.516	bb	261.1	
17	17	151117_S_6	Analyte		1.67	1808.196	7552.594	0.239	bb	118.7	
18	18	151117_ QC_A_2	QC	30.000	1.67	919.512	12742.463	0.072	bb	32.5	8.5
19	19	151117_ QC_B_2	QC	170.000	1.67	4678.028	11741.281	0.398	bb	200.6	18.0
20	20	151117_ QC_C_2	QC	250.000	1.67	6176.616	11770.134	0.525	bb	265.7	6.3
21	21	151117_ QC_A_3	QC	30.000	1.67	863.481	12417.033	0.070	bb	31.2	4.0
22	22	151117_ QC_B_3	QC	170.000	1.67	4505.483	12984.740	0.347	bb	174.1	2.4
23	23	151117_ QC_C_3	QC	250.000	1.67	6123.393	12252.269	0.500	bb	252.9	1.1

Dataset: C:\MassLynx\MAHM_13Jul15.PRO\LINEALITY_ANALYSIS_11302017.qld

Last Altered: Thursday, November 30, 2017 15:04:17 Central Standard Time (Mexico)
Printed: Thursday, November 30, 2017 15:07:55 Central Standard Time (Mexico)

Sample Name: 151117_STD_1

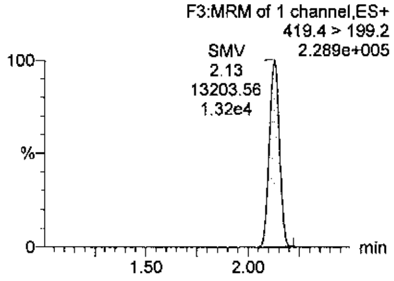

Sample Name: 151117_STD_2

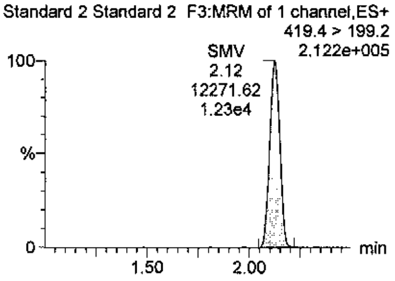

Sample Name: 151117_STD_3

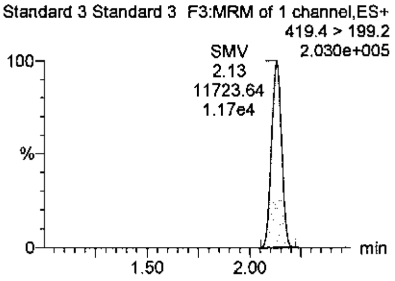

Sample Name: 151117_STD_4

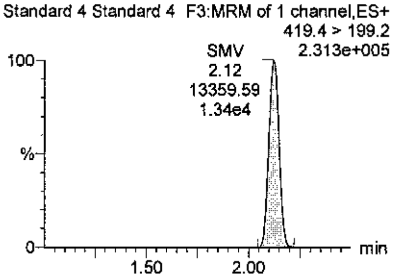

Sample Name: 151117_STD_5

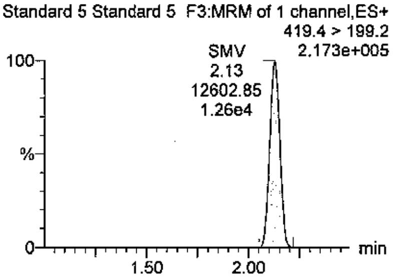

Sample Name: 151117_STD_6

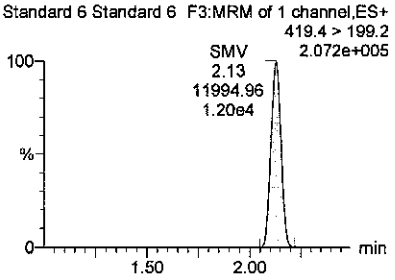

Sample Name: 151117_STD_7

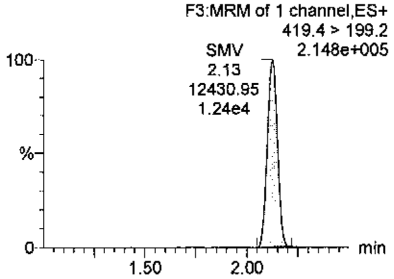

Sample Name: 151117_Blank_22

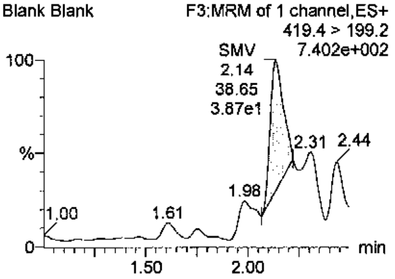

Sample Name: 151117_ QC_A_1

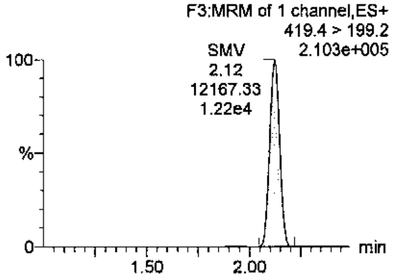

Sample Name: 151117_ QC_B_1

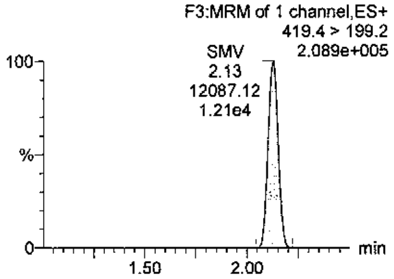

Sample Name: 151117_ QC_C_1

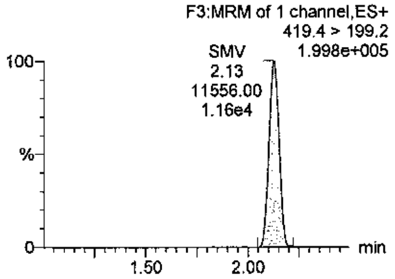

Sample Name: 151117_S_1

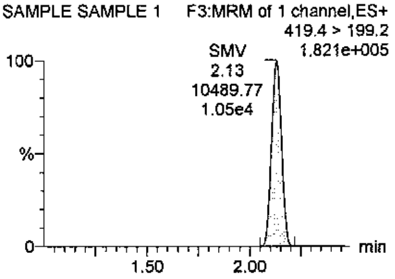

Quantify Compound Report **MassLynx 4.1 SCN 843**
Cuantificación de Antiretrovirales en Leche Materna

Dataset: C:\MassLynx\MAHM_13Jul15.PRO\LINEALITY_ANALYSIS_11302017.qld

Last Altered: Thursday, November 30, 2017 15:04:17 Central Standard Time (Mexico)
Printed: Thursday, November 30, 2017 15:07:55 Central Standard Time (Mexico)

Sample Name: 151117_S_2
SAMPLE SAMPLE 2 F3:MRM of 1 channel,ES+
419.4 > 199.2
1.399e+005

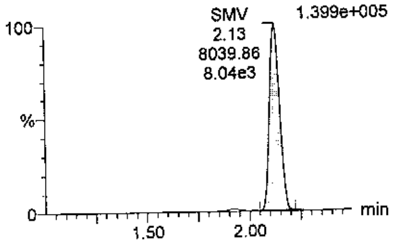

Sample Name: 151117_S_3
SAMPLE SAMPLE 3 F3:MRM of 1 channel,ES+
419.4 > 199.2
2.055e+005

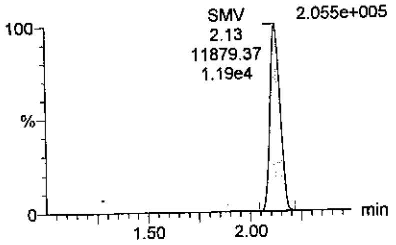

Sample Name: 151117_S_4
SAMPLE SAMPLE 4 F3:MRM of 1 channel,ES+
419.4 > 199.2
8.298e+004

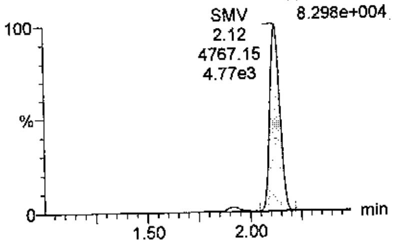

Sample Name: 151117_S_5
SAMPLE SAMPLE 5 F3:MRM of 1 channel,ES+
419.4 > 199.2
1.320e+005

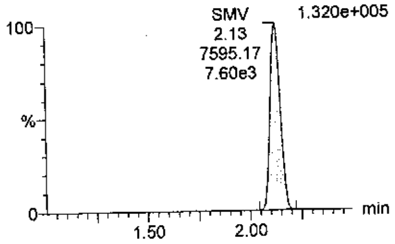

Sample Name: 151117_S_6
SAMPLE SAMPLE 6 F3:MRM of 1 channel,ES+
419.4 > 199.2
1.307e+005

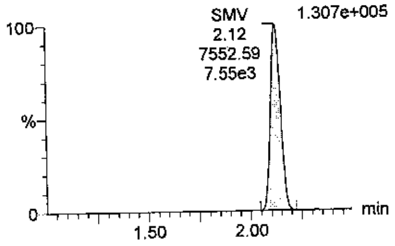

Sample Name: 151117_ QC_A_2
F3:MRM of 1 channel,ES+
419.4 > 199.2
2.216e+005

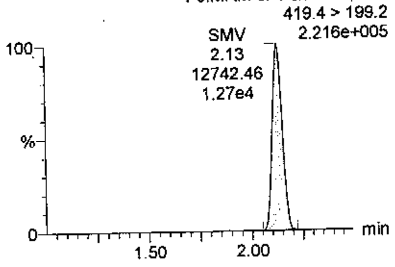

Sample Name: 151117_ QC_B_2
F3:MRM of 1 channel,ES+
419.4 > 199.2
2.039e+005

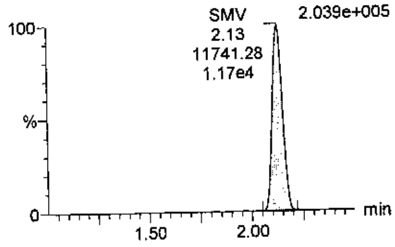

Sample Name: 151117_ QC_C_2
F3:MRM of 1 channel,ES+
419.4 > 199.2
2.041e+005

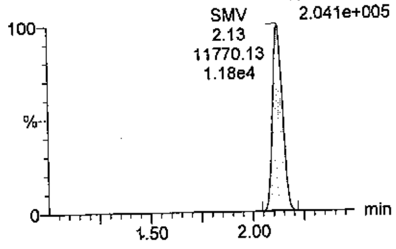

Sample Name: 151117_ QC_A_3
F3:MRM of 1 channel,ES+
419.4 > 199.2
2.162e+005

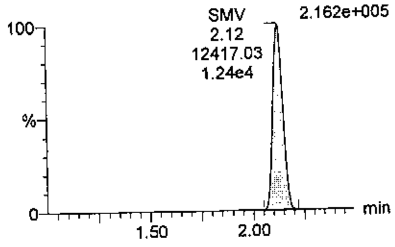

Sample Name: 151117_ QC_B_3
F3:MRM of 1 channel,ES+
419.4 > 199.2
2.273e+005

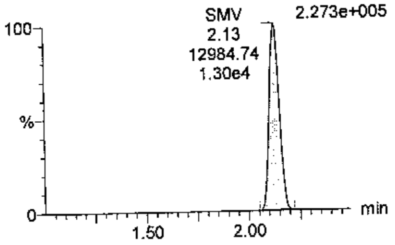

Sample Name: 151117_ QC_C_3
F3:MRM of 1 channel,ES+
419.4 > 199.2
2.139e+005

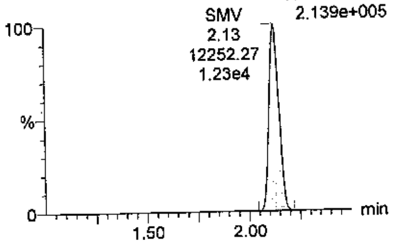

Cuantificación de Antiretrovirales en Leche Materna

Dataset: C:\MassLynx\MAHM_13Jul15.PRO\LINEALITY_ANALYSIS_11302017.qld

Last Altered: Thursday, November 30, 2017 15:04:17 Central Standard Time (Mexico)
Printed: Thursday, November 30, 2017 15:07:55 Central Standard Time (Mexico)

	#	Name	Type	Std. Conc	RT	Area	IS Area	Response	Primar...	ng/mL	%Dev
1	1	151117_STD_1	Standard	1.000	2.13	13203.555		13203.555	bb	1.1	5.5
2	2	151117_STD_2	Standard	1.000	2.12	12271.620		12271.620	bb	1.0	-1.9
3	3	151117_STD_3	Standard	1.000	2.13	11723.644		11723.644	bb	0.9	-6.3
4	4	151117_STD_4	Standard	1.000	2.12	13359.588		13359.588	bb	1.1	6.8
5	5	151117_STD_5	Standard	1.000	2.13	12602.851		12602.851	bb	1.0	0.7
6	6	151117_STD_6	Standard	1.000	2.13	11994.957		11994.957	bb	1.0	-4.1
7	7	151117_STD_7	Standard	1.000	2.13	12430.945		12430.945	bb	1.0	-0.7
8	8	151117_Blank_22	Blank	1.000	2.14	38.652		38.652	bb	0.0	-99.7
9	9	151117_ QC_A_1	QC	1.000	2.12	12167.331		12167.331	MM	1.0	-2.8
10	10	151117_ QC_B_1	QC	1.000	2.13	12087.120		12087.120	bb	1.0	-3.4
11	11	151117_ QC_C_1	QC	1.000	2.13	11556.002		11556.002	bb	0.9	-7.6
12	12	151117_S_1	Analyte	1.000	2.13	10489.766		10489.766	bb	0.8	-16.2
13	13	151117_S_2	Analyte	1.000	2.13	8039.864		8039.864	bb	0.6	-35.7
14	14	151117_S_3	Analyte	1.000	2.13	11879.370		11879.370	bb	0.9	-5.1
15	15	151117_S_4	Analyte	1.000	2.12	4767.146		4767.146	bb	0.4	-61.9
16	16	151117_S_5	Analyte	1.000	2.13	7595.172		7595.172	bb	0.6	-39.3
17	17	151117_S_6	Analyte	1.000	2.12	7552.594		7552.594	bb	0.6	-39.6
18	18	151117_ QC_A_2	QC	1.000	2.13	12742.463		12742.463	bb	1.0	1.8
19	19	151117_ QC_B_2	QC	1.000	2.13	11741.281		11741.281	bb	0.9	-6.2
20	20	151117_ QC_C_2	QC	1.000	2.13	11770.134		11770.134	bb	0.9	-5.9
21	21	151117_ QC_A_3	QC	1.000	2.12	12417.033		12417.033	bb	1.0	-0.8
22	22	151117_ QC_B_3	QC	1.000	2.13	12984.740		12984.740	bb	1.0	3.8
23	23	151117_ QC_C_3	QC	1.000	2.13	12252.269		12252.269	bb	1.0	-2.1
