# Supplementary material for: Simultaneous quantification of four antiretroviral drugs in breast milk samples from HIV-positive women by an ultra-high performance liquid chromatography tandem mass spectrometry (UPLC-MS/MS) method
Source: PLoS One. 2018 Jan 19;13(1):e0191236. doi: 10.1371/journal.pone.0191236 (PMC5774716; doi:10.1371/journal.pone.0191236)
Supplement: S3 Fig — A) Blank with IS; B) LLOQ of LPV with IS; C) LQC of LPV with IS; D) MQC of LPV with IS; E) HQC of LPV with IS; and F) Blank of breast milk (.TIF). (PDF) [file pone.0191236.s004.pdf]

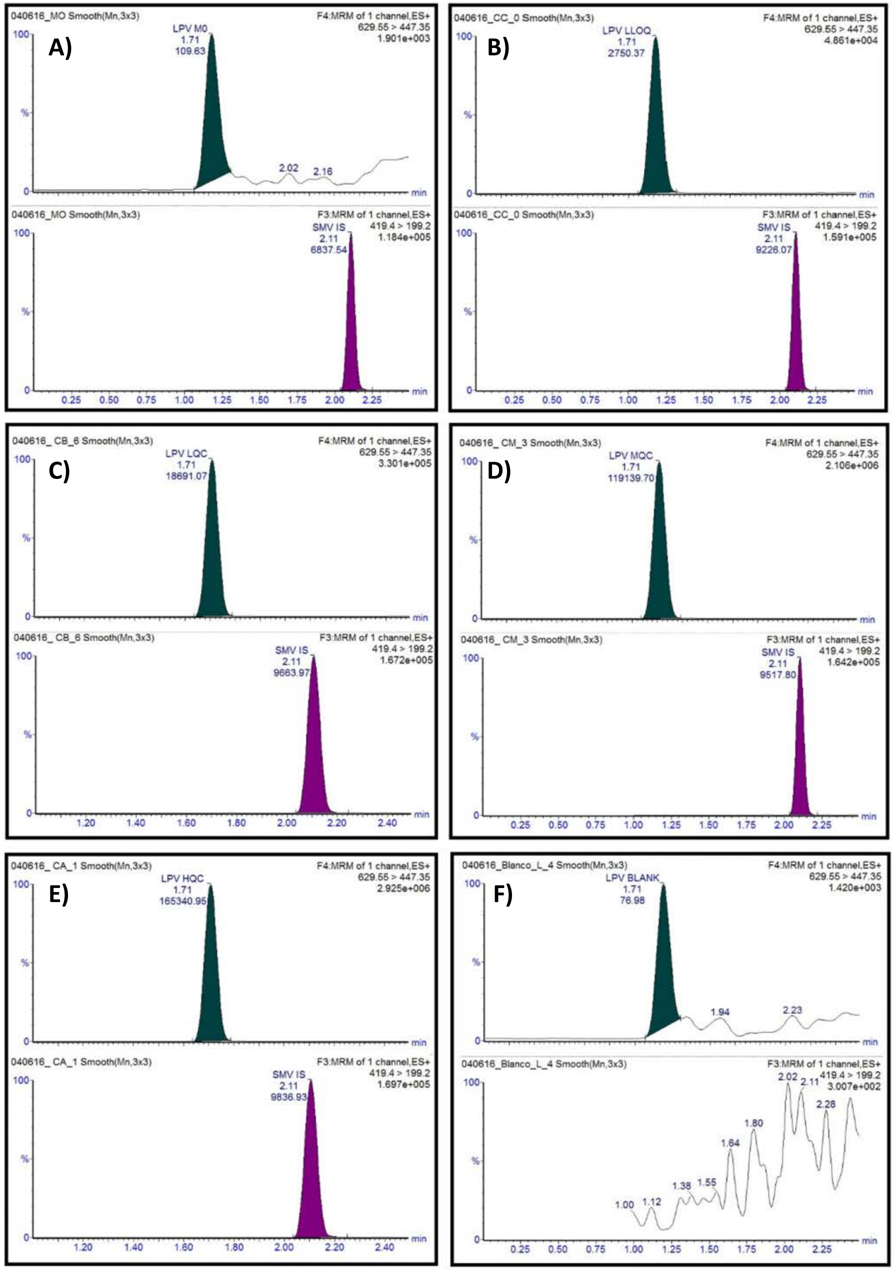

S3 Fig. Representative chromatograms of LPV extracted from breast milk. A) Blank with IS; B) LLOQ of LPV with IS; C) LQC of LPV with IS; D) MQC of LPV with IS; E) HQC of LPV with IS; and F) Blank of breast milk (.TIF)
