# Supplementary material for: Simultaneous quantification of four antiretroviral drugs in breast milk samples from HIV-positive women by an ultra-high performance liquid chromatography tandem mass spectrometry (UPLC-MS/MS) method
Source: PLoS One. 2018 Jan 19;13(1):e0191236. doi: 10.1371/journal.pone.0191236 (PMC5774716; doi:10.1371/journal.pone.0191236)
Supplement: S5 Fig — Total run time was 2.5 minutes, with an ACQUITY UPLC BEH C 18 column, formic acid and ACN gradient as mobile phase (.TIF). (PDF) [file pone.0191236.s006.pdf]

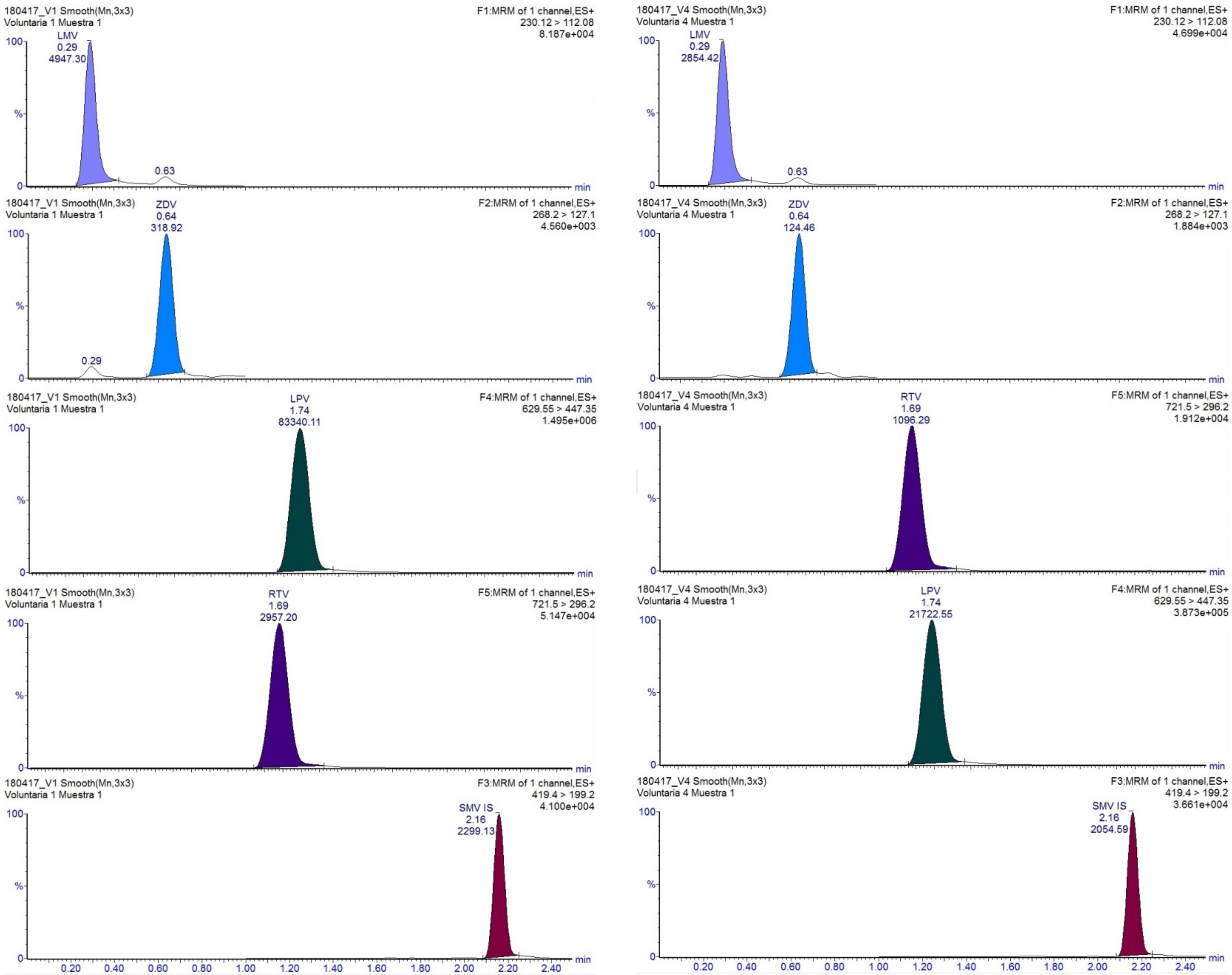

S5 Fig Representative chromatograms of separation and retention time (minutes) of each ARV drug and internal standard from two HIV-positive woman breast milk sample: 0.29, 0.64, 1.69, 1.74 and 2.16 for LMV, ZDV, RTV, LPV, and SMV. Total run time was 2.5 minutes, with an ACQUITY UPLC BEH C 18 column, formic acid and ACN gradient as mobile phase. **(.TIF)**
