# Supplementary material for: Simultaneous quantification of four antiretroviral drugs in breast milk samples from HIV-positive women by an ultra-high performance liquid chromatography tandem mass spectrometry (UPLC-MS/MS) method
Source: PLoS One. 2018 Jan 19;13(1):e0191236. doi: 10.1371/journal.pone.0191236 (PMC5774716; doi:10.1371/journal.pone.0191236)
Supplement: S1 Table — (PDF) [file pone.0191236.s007.pdf]

S1 Table. Accuracy and precision for the quantification of antiretroviral in breast milk

STDs (ng/mL)	Inter-day (measured concentration)				Intra-day (measured concentration)			
	Mean	SD	Precision (%CV)	Accuracy (%)	Mean	SD	Precision (%CV)	Accuracy (%)
**ZDV (nominal concentration)**								
**12.5**	13.55	1.05	7.77	90.87	12.64	0.68	5.38	95.62
**25**	24.31	2.38	9.80	93.26	25.99	0.90	3.49	95.93
**50**	44.35	2.05	4.63	88.71	46.88	0.75	1.60	93.77
**125**	125.02	15.19	12.15	91.17	121.41	0.90	0.74	97.13
**250**	269.07	12.70	4.72	92.38	239.32	15.76	6.58	95.68
**500**	504.75	53.11	10.52	91.64	515.98	5.60	1.08	96.80
**750**	745.00	81.38	10.92	92.01	763.86	46.38	6.07	95.83
**LMV (nominal concentration)**								
**50**	49.36	0.35	0.724	98.74	49.46	1.79	3.62	97.60
**100**	99.59	1.50	1.50	98.74	101.28	0.39	0.39	98.71
**200**	198.89	5.02	2.52	98.28	203.56	11.86	5.82	96.09
**500**	508.83	17.31	3.40	97.50	482.51	2.69	0.55	96.50
**1000**	1061.84	54.43	5.12	93.82	994.60	43.00	4.32	96.99
**2000**	1916.55	247.68	12.92	91.25	2027.27	52.84	2.60	97.54
**3000**	2938.32	160.11	5.44	95.23	2994.77	152.78	5.10	96.06
**LPV (nominal concentration)**								
**100**	96.79	5.50	5.69	94.72	98.86	2.10	2.13	98.01
**200**	199.86	7.50	3.75	97.16	197.84	13.87	7.01	95.10
**400**	425.90	42.50	9.98	89.73	414.69	29.19	7.03	93.42
**1000**	1017.91	62.55	6.14	94.64	988.33	85.97	8.69	93.92
**2000**	1931.80	65.48	3.38	96.60	2027.88	126.27	6.22	94.71
**4000**	3897.31	233.05	5.97	95.26	4038.46	106.46	2.63	98.28
**6000**	6129.07	255.49	4.16	96.31	5948.12	423.47	7.11	95.00
**RTV (nominal concentration)**								
**5**	5.83	0.35	6.31	87.45	5.77	0.21	3.80	84.50
**10**	9.06	0.35	3.92	90.70	9.39	0.65	6.96	93.93
**20**	19.71	1.52	7.74	94.55	17.91	0.48	2.69	89.55
**50**	47.86	1.45	3.04	95.73	48.59	1.51	3.11	97.18
**100**	96.35	1.03	1.07	96.37	96.00	2.69	2.80	96.00
**200**	209.74	11.08	5.28	94.24	205.11	11.45	5.58	94.94
**300**	310.08	19.07	6.15	96.23	321.22	15.12	4.70	92.93
