# Supplementary material for: Simultaneous quantification of four antiretroviral drugs in breast milk samples from HIV-positive women by an ultra-high performance liquid chromatography tandem mass spectrometry (UPLC-MS/MS) method
Source: PLoS One. 2018 Jan 19;13(1):e0191236. doi: 10.1371/journal.pone.0191236 (PMC5774716; doi:10.1371/journal.pone.0191236)
Supplement: S2 Table — (PDF) [file pone.0191236.s008.pdf]

S2 Table. Demographics and clinical and ART individual data of HIV-positive women included in this study from National Institute of Perinatology, Mexico City, Mexico.

**Patient**	**Variable**					
	Age (years)	Weight (Kg)	Height (cm)	gestational age (weeks)	CD4+ lymphocyte (cell/mm3) data	ARV regimen
Patient 1	23	60,4	150	38,8	607	LPV/RTV (200/50 bid) and ZDV/LMV (300/150 bid)
Patient 2	27	74,6	nd	37,0	nd	as prophylaxis ZDV/LMV (300/150 tid)<br>LPV/RTV (200/50 tid)
Patient 3	24	56,65	159	37,3	1593	Tenofovir/Emtricitabine (200/300 mg qd), Raltegravir (400 mg bid)<br>as prophylaxis LPV/RTV (200/50 mg tid)<br>ZDV/LMV (300/150 tid)
Patient 4	32	101	170	37,2	105	Abacavir/LMV<br>as prophylaxis LPV/RTV (200/50 mg tid) and ZDV/LMV (300/150 mg tid)
Patient 5	32	53,65	153	38,5	749	LPV/RTV (200/50 mg bid) and Tenofovir/Emtricitabine (200/300 mg qd)
Patient 6	27	60,85	157	37,6	647	Efavirenz and Kivexa; as prophylaxis ZDV/LMV, (300/150 tid)
Patient 7	36	66,7	157	39,2	447	Tenofovir/emtricitabine (300/200 bid) and Raltegravir (400 g qd)
Patient 8	38	82	153	37,6	nd	Tenofovir/Emtricitabine/ (200/300 mg qd)<br>as prophylaxis LPV/RTV (200/50 tid).
Patient 9	31	65,1	159	nd	386	Emtricitabine/Tenofovir y Raltegravir (400 mg bid)
**Mean**	**30**	**68,99**	**157,25**	**37,9**	**647,71**	
**SD**	**5,14**	**14,88**	**6,06**	**0,81**	**467,04**	

qd: once a day; bid: two times a day; tid: three times a day
nd: not data
