# Supplementary material for: Simultaneous quantification of four antiretroviral drugs in breast milk samples from HIV-positive women by an ultra-high performance liquid chromatography tandem mass spectrometry (UPLC-MS/MS) method
Source: PLoS One. 2018 Jan 19;13(1):e0191236. doi: 10.1371/journal.pone.0191236 (PMC5774716; doi:10.1371/journal.pone.0191236)
Supplement: S3 Table — (PDF) [file pone.0191236.s009.pdf]

S3 Table. Antiretroviral levels in breast milk samples from HIV-positive women.

Patient	ARV measured concentration (ng/mL)			
	LMV	ZDV	LPV	RTV
Patient 1	574.22	99.54	5772.98	387.92
Patient 2	1148.11	65.19	1143.18	119.15
Patient 3	503.02	21.88	14295.83	382.34
Patient 4	725.04	45.29	1518.65	146.22
Patient 5	308,71	17,87	4771,57	402,94
Patient 6	143,26	100,71	nq	nq
Patient 7	188,14	87,80	2520,79	98,16
Patient 8	176,36	19,85	2845,59	261,05
Patient 9	272,60	447,81	1234,70	118,71
**mean**	**448,83**	**100,66**	**4262,91**	**239,56**
**%CV**	**73,48**	**132,56**	**102,89**	**56,31**

nq: not quantifiable
